# Weighting and indirect effects identify keystone species in food webs

**DOI:** 10.1111/ele.12638

**Published:** 2016-06-27

**Authors:** Lei Zhao, Huayong Zhang, Eoin J. O'Gorman, Wang Tian, Athen Ma, John C. Moore, Stuart R. Borrett, Guy Woodward

**Affiliations:** ^1^ Research Center for Engineering Ecology and Nonlinear ScienceNorth China Electric Power UniversityBeijing102206China; ^2^ Department of Life SciencesImperial College LondonSilwood Park Campus, Buckhurst Road, AscotBerkshireSL5 7PYUK; ^3^ School of Electronic Engineering and Computer ScienceQueen Mary University of LondonMile End RoadLondonE1 4NSUK; ^4^ Department of Ecosystem Science and SustainabilityColorado State UniversityFort CollinsCO80523USA; ^5^ Natural Resource Ecology LaboratoryColorado State UniversityFort CollinsCO80523USA; ^6^ Department of Biology and Marine BiologyUniversity of North Carolina WilmingtonWilmingtonNC28403USA; ^7^ Duke Network Analysis CenterDuke UniversityDurhamNC27708USA

**Keywords:** Carbon flux, centrality, energy budget, quantitative food web, robustness, secondary extinction, sequential deletion, species loss

## Abstract

Species extinctions are accelerating globally, yet the mechanisms that maintain local biodiversity remain poorly understood. The extinction of species that feed on or are fed on by many others (i.e. ‘hubs’) has traditionally been thought to cause the greatest threat of further biodiversity loss. Very little attention has been paid to the strength of those feeding links (i.e. link weight) and the prevalence of indirect interactions. Here, we used a dynamical model based on empirical energy budget data to assess changes in ecosystem stability after simulating the loss of species according to various extinction scenarios. Link weight and/or indirect effects had stronger effects on food‐web stability than the simple removal of ‘hubs’, demonstrating that both quantitative fluxes and species dissipating their effects across many links should be of great concern in biodiversity conservation, and the potential for ‘hubs’ to act as keystone species may have been exaggerated to date.

## Introduction

Biodiversity loss is a major threat to Earth's ecosystems (Barnosky *et al*. 2011) and it is crucial to identify and conserve influential ‘keystone’ species or nodes, whose loss would cause the cascading extinctions of many other species (Dunne *et al*. 2002; Jordán 2009). Theoretical and empirical approaches to studying the importance of interactions among species in maintaining biodiversity recognise that there is a bi‐directional component to every interaction that gauges the separate impacts of one species on the dynamics of another (May 1972; Tang *et al*. 2014). For every direct interaction, the consumer will have a negative effect on the resource and the resource will have a positive effect on the consumer. Simple binary measures of interaction strength have been used to identify ‘hubs’, i.e. highly connected nodes with a high‐degree centrality (Dunne *et al*. 2002; Memmott *et al*. 2004; Dunne & Williams 2009). Selective removal of nodes with the most trophic links in a network typically causes more secondary extinctions than random removal of nodes (Dunne *et al*. 2002; Memmott *et al*. 2004; Dunne & Williams 2009). However, this index of node importance based on degree centrality ignores two major components of food webs: the strength or weight of the links and indirect effects (Scotti *et al*. 2007). This can lead to an inaccurate ranking of species importance, such that removing the most‐connected nodes does not necessarily identify the most destructive extinction sequence (Allesina & Pascual 2009).

There has been a tradition of binary descriptions in many network studies, reflecting the relative ease of data collection, but there is a growing appreciation that this qualitative network structure is often uninformative (Jordán *et al*. 2006). An increasing number of studies now consider weighted networks in ecology (Jordán *et al*. 2006; Borrett 2013; Ulanowicz *et al*. 2014), which can dramatically alter the conclusions about node importance (Scotti *et al*. 2007; Jordán 2009). In many quantitative food webs, link weights (i.e. the strength of trophic interactions) have been estimated based on the biomass, numbers of individuals or carbon flows between species or compartments (Moore *et al*. 1993; Jordán *et al*. 2006; Borrett 2013). These quantitative approaches are arguably more robust than binary methods, but are not without criticism (Paine 1980). For example, controlled removal studies have demonstrated empirically that material flow does not always translate directly to the impacts that interaction strength purports to capture (Woodward *et al*. 2005).

Indirect effects describe the impact of one species on another that is mediated by a third species (Montoya *et al*. 2009), and their importance for the maintenance of structure, stability and biodiversity in food webs has been increasingly emphasised (Bukovinszky *et al*. 2008; Woodward *et al*. 2008; Sanders *et al*. 2013). Both empirical (Sanders *et al*. 2013) and theoretical (Stouffer & Bascompte 2011; Säterberg *et al*. 2013) studies show that secondary extinctions can occur even when a species is more than one trophic link away from the primary extinction. Recent studies that used a static structural approach showed that direct and indirect indices provide quite different rankings of node importance in networks (Scotti *et al*. 2007; Jordán 2009) and we do not know which of the indices performs best at identifying keystone species for maintaining biodiversity.

There are two main approaches to simulating the cascading extinctions that occur after primary removals: topological and dynamical analyses (Eklöf & Ebenman 2006). Both approaches have strengths and weaknesses: the former considers only binary network structure and so is easier to parameterise, whereas the latter takes into account both link structure and changes in species abundance through time (Curtsdotter *et al*. 2011). In topological models, secondary extinctions emerge from bottom‐up cascades (Eklöf & Ebenman 2006). In a natural system, the loss of species can also cause top‐down extinction cascades (Elmhagen & Rushton 2007; Säterberg *et al*. 2013), meaning that the full range of indirect effects is not covered and food‐web robustness is often overestimated (Curtsdotter *et al*. 2011). We chose the dynamical approach here and we simulated natural communities using parameter values derived from empirical data, which should provide more realistic outcomes than can be derived from simulating purely artificial communities (Curtsdotter *et al*. 2011). Furthermore, both top‐down and bottom‐up effects are possible in the dynamical approach, therefore extinction cascades in both directions could be detected. We expect the assessment of node importance indices using the dynamical approach should bring new insights into the magnitude and extent of secondary extinctions.

We compared the performance of four different ranking scenarios (considering direct effects only, both direct and indirect effects, weighted direct effects only and weighted direct and indirect effects) at identifying taxa that maintain biodiversity. Considering the importance of link weight and indirect effects, we expect the nodes with large carbon flux or dissipating their effects across many nodes would be influential. We hypothesised that the indices considering weighted links and/or indirect effects would perform better than the more traditional measures: i.e. more secondary extinctions will be caused in deletion sequences ordered by link weight, direct plus indirect effects or both.

## Materials and Methods

### Quantitative food webs

We analysed 20 of the 50 aquatic food webs from a recently published database (see Table 1; Salas & Borrett 2011; Borrett 2013). The extraction criteria were as follows: (1) small networks (containing no more than 10 nodes) which are easily collapsed were excluded; (2) each dataset was from a distinct study system to avoid pseudoreplication of similar networks from the same location (e.g. we randomly chose one web from Florida Bay Dry Season and Florida Bay Wet Season).

**Table 1:** Original names and structural properties of the 20 empirical food webs examined in the study

Food web	Original name	*S* a	*C* b	*C*_*w*_ c
Bothnian Bay	Bothnian Bay	12	0.222	0.184
Baltic Sea	Baltic Sea	15	0.173	0.184
Ems Estuary	Ems Estuary	15	0.196	0.169
Swartkops	Swartkops Estuary	15	0.169	0.121
Crystal River	Crystal River (control)	21	0.186	0.070
Benguela	Northern Benguela Upwelling	24	0.208	0.101
Neuse Estuary	Neuse Estuary (late summer 1998)	30	0.138	0.062
Georges Bank	Georges Bank	31	0.354	0.162
Gulf of Maine	Gulf of Maine	31	0.345	0.148
Narragansett	Narragansett Bay	32	0.154	0.093
Atlantic Bight	Middle Atlantic Bight	32	0.366	0.156
New England	Southern New England Bight	33	0.347	0.154
Chesapeake	Chesapeake Bay	36	0.094	0.068
St. Marks	St. Marks Seagrass, site 1 (Feb.)	51	0.103	0.086
Graminoids	Graminoids (wet)	66	0.182	0.033
Cypress	Cypress (wet)	68	0.118	0.060
Lake Oneida	Lake Oneida (pre‐ZM)	74	0.223	0.072
Bay of Quinte	Bay of Quinte (pre‐ZM)	74	0.211	0.056
Mangroves	Mangroves (wet)	94	0.152	0.036
Florida Bay	Florida Bay (wet)	125	0.124	0.029

a Number of taxa.

b Binary directed connectance, *L/S* ^2^; where *L* is the number of trophic links.

c weighted directed connectance (see Materials and Methods for calculation method).

The data for each food web include a list of taxa, the carbon biomass of each taxon (g C m^−2^), the carbon per unit time of import, export and respiration of each taxon (g C m^−2^ day^−1^) and the carbon flux between a pair of taxa (g C m^−2^ day^−1^). The dataset was archived in the ‘enaR’ package in R (Borrett & Lau 2014). Nodes represent species, trophic guilds, functional groups or non‐living components of the system in which matter is stored. Initially unbalanced food webs, i.e. energy entering a taxon does not exactly balance the output, were balanced using the AVG2 algorithm using established procedures in Matlab 7.12.0 (Allesina & Bondavalli 2003). Our focal food webs exhibit a wide range of network complexity, indicated by taxon richness (*S *= 12–125), binary directed connectance (*C* = 0.094–0.366) and weighted directed connectance (*C*_*w*_ = 0.029–0.184), but all of these were within the range reported for other recently described catalogues (see Table 1; cf. Williams & Martinez 2000; Ings *et al*. 2009). Binary directed connectance is a qualitative descriptor based on binary networks, which measures the proportion of possible links between taxa that are realised; weighted directed connectance *C*_*w*_ is a quantitative descriptor based on Shannon's entropy (Banašek‐Richter *et al*. 2009; see Appendix S1 for details).

### Food‐web dynamics

The model was constructed based on energy budgets that index the carbon fluxes entering and leaving each taxon. The imports and exports *via* animal migration and water flows are considered to be in balance and not to influence the food‐web dynamics, similar to many other dynamical models (Moore *et al*. 1993; Hudson & Reuman 2013). In general, the taxa in the system can be divided into four categories (see Fig. S1): producers, consumers, decomposers and non‐living compartments (i.e. detritus).

The change in biomass of producers can be described as:

(1) $$\frac{dB_{i}}{dt}=r_{i}B_{i}G_{i}-\sum_{j=herbi}\Phi_{ij}B_{j}-d_{i}B_{i}$$

Here, ‘*herbi*’ are herbivorous taxa, *r* is the maximum specific or intrinsic growth rate and *G*_*i*_ is the growth model, following $G_{i}=1-\sum_{j=pro}B_{j}/K$. Here, ‘*pro*’ are producer taxa and *K* is the carrying capacity. The value of *K* is considered as the total initial producer biomass in the community multiplied by a term 10^*k*0^. The carrying capacity coefficient *k*_0_ was set to follow the distribution U[0, 3] (after Hudson & Reuman 2013). Φ_*ij*_ is the functional response when taxon *j* consumes taxon *i* (see below for more details), and *d* is the specific death rate. The biomass of producer taxon *i* is increased by photosynthesis and decreased by intertaxon competition, consumption and non‐predatory death.

The change in biomass of consumers (including herbivores and predators) can be depicted as:

(2) $$\frac{dB_{i}}{dt}=\sum_{j=res}a_{i}\Phi_{ji}B_{i}-\sum_{j=pred}\Phi_{ij}B_{j}-x_{i}B_{i}$$

Here, ‘*res*’ means resource taxon, ‘*pred*’ means predator taxon, *a* is the assimilation efficiency and *x* is the respiration rate. The biomass of consumer taxon *i* is increased by assimilation of consumed resources and decreased by predation and respiration.

The change in biomass of decomposers can be depicted as:

(3) $$\frac{dB_{i}}{dt}=\sum_{j=det}a_{i}\Phi_{ji}B_{i}-\sum_{j=pred}\Phi_{ij}B_{j}-x_{i}B_{i}$$

Here, ‘*det*’ are detrital taxa. The biomass of decomposer taxon *i* is increased by assimilation of consumed detritus and decreased by predation and respiration.

In some food webs, detritus has been divided into separate compartments. For example, there are three detrital taxa in the Florida Bay ecosystem: water particulate organic carbon (POC), benthic POC and dissolved organic carbon. The change in biomass of each detrital taxa can be described as:

(4) $$\frac{dB_{i}}{dt}=\sum_{j=pro}p_{ji}d_{j}B_{j}+\sum_{j=con}\left(p_{ji}e_{j}B_{j}\sum_{k=res}\Phi_{kj}\right)+\sum_{j=det}c_{ji}B_{j}-\sum_{j=dec}\Phi_{ij}B_{j}-\sum_{j=det}c_{ij}B_{i}$$

Here, ‘*con*’ are consumer taxa and ‘*dec*’ are decomposer taxa, *p*_*ji*_ is the proportion of converted detritus *i* to the total amount of detritus converted from taxon *j*,* e = *(1 − *a*) is the egestion rate and *c*_*ji*_ is the conversion coefficient from detrital taxon *j* to detrital taxon *i*. Here we consider that the amount of faeces, i.e. the unassimilated fraction of prey killed, is proportional to the amount of predation (Moore *et al*. 1993; de Ruiter *et al*. 1995; Moore & de Ruiter 2012). The biomass stored in detrital taxon *i* is increased by the dead bodies of producer taxa, the faeces of consumer taxa and the conversion from other detritus, and decreased by consumption of decomposer taxa and conversion into other detritus. The meanings and calculations of the parameters listed above (except the functional response Φ which was given below) can be found in Table 2.

**Table 2:** Details of the parameters used in the model

Symbol	Meaning	Value	Unit
*r*_*i*_	Maximum specific or intrinsic growth rate	$\left(GPP_{i}-R_{i}\right)/\left[B_{i}\left(1-1/10^{k_{0}}\right)\right]$	day^−1^
*K*	Carrying capacity	$10^{k_{0}}\sum_{j=pro}B_{i}$	g C m^−2^
*d*_*i*_	Natural specific death rate	$\sum_{j=\det}F_{ij}/B_{i}$	day^−1^
*a*_*i*_	Assimilation efficiency	$1-\sum_{j=\det}F_{ij}/\sum_{j=res}F_{ji}$	Proportion (unitless)
*x*_*i*_	Respiration rate	*R*_*i*_/*B*_*i*_	day^−1^
*p*_*ji*_	Proportion of converted detritus *i* in all the converted detritus from producer or consumer taxon *j*	$F_{ji}/\sum_{k=\det}F_{jk}$	Proportion (unitless)
*e*_*i*_	Egestion rate	$1-a_{i}$	Proportion (unitless)
*c*_*ji*_	Conversion coefficient from detritus *j* to detritus *i*	*F*_*ji*_/*B*_*j*_	day^−1^

Our data (see Table 1) contain the values of *GPP*_*i*_ (gross primary production), *R*_*i*_ (respiration), *B*_*i*_ (biomass) and *F*_*ij*_ (carbon flux when taxon *j* consumes taxon *i*). *k*_0_ is an undetermined parameter. Considering that carrying capacity *K* was within three orders of magnitude of total primary producer biomass in the community being simulated (Hudson & Reuman 2013), we assumed *k*_0_ follows the distribution U[0, 3]. We ran 1000 separate simulations for each food web, using different values of *k*_0_, chosen randomly from this distribution.

### Functional forms

The functional response Φ_*ij*_ was set to follow either a nonlinear form or a linear form. The nonlinear form was set as follows (see Hudson & Reuman 2013):

(5) $$\Phi_{ij}=\frac{y_{j}\omega_{ij}B_{i}^{h}}{H_{j}^{h}+q_{j}B_{j}H_{j}^{h}+\sum_{k=res}\omega_{kj}B_{k}^{h}}$$

Here, *y*_*j*_ is the maximum consumption rate of taxon *j* and ω_*ij*_ is the preference of taxon *j* for taxon *i*. For a consumer *j*, $\omega_{ij}\propto F_{ij}/B_{i}^{h}$. *F*_*ij*_ is the carbon flux from taxon *i* to taxon *j*, which was contained in the empirical data. Given that $\sum_{i=res}\omega_{ij}=1$, we can calculate ω_*ij*_ as:

(6) $$\omega_{ij}=\frac{F_{ij}/B_{i}^{h}}{\sum_{k=res}F_{kj}/B_{k}^{h}}$$

*H*_*j*_ is the half‐saturation density, which was one order of magnitude either side of the mean of all biomasses in the community being simulated (Hudson & Reuman 2013). That means $H_{j}=10^{b}\bar{B}$. Here, *b* is a coefficient following the distribution U[−1, 1]. *q*_*j*_ is the predator interference coefficient, which was randomly chosen from 0 to 100 (Hudson & Reuman 2013). *h* is the hill exponent that regulates the shape of the curve from Holling Type II (*h* = 1) to Holling Type III (*h* = 2). We chose the value of *h* randomly from 1 to 2.

Notice that *F*_*ij*_ = Φ_*ij*_ *B*_*j*_, combining eqn 5, and we can calculate the value of *y*_*j*_ by:

(7) $$y_{j}=\frac{F_{ij}\left(H_{j}^{h}+q_{j}B_{j}H_{j}^{h}+\sum_{k=res}\omega_{kj}B_{k}^{h}\right)}{\omega_{ij}B_{i}^{h}B_{j}}$$

We ran 1000 simulations for each food web. The values of parameter *b*,* q* and *h* for each simulation were chosen randomly from their ranges, i.e. U[−1, 1] for *b*, U[0, 100] for *q* and U[1, 2] for *h*.

To increase the generality of our model, we also applied the linear functional response, i.e. the Holling Type I. The linear form of the functional response Φ_*ij*_ is as follows:

(8) $$\Phi_{ij}=f_{ij}B_{i}$$

Here, *f*_*ij*_ is the feeding rate coefficient when taxon *j* consumes taxon *i*. The value of *f*_*ij*_ can be obtained by:

(9) $$f_{ij}=\frac{F_{ij}}{B_{i}B_{j}}$$

### Sequential node deletions

Following the framework of Scotti *et al*. (2007), but using the whole food‐web dynamical model, we compared four different rankings based on the presence or absence of information on indirect effects and weighted links. Here, we ordered nodes by their: (1) maximum unweighted direct effect (Max.D); (2) maximum unweighted direct plus indirect effect (Max.DI); (3) maximum weighted direct effect (Max.wD) and (4) maximum weighted direct plus indirect effect (Max.wDI). Unweighted direct effect is defined as the degree centrality of a node (i.e. the number of its direct neighbours including both consumers and resources), whereas weighted direct effect of a node is defined as the total amount of its inwards and outwards carbon fluxes. The unweighted direct plus indirect effect is the mean of effects originating from one taxon in a binary network, whereas weighted direct plus indirect effect has the same meaning but in a weighted network.

The method for quantifying the direct plus indirect effects has been used in both undirected (Jordán *et al*. 2006; Jordán 2009) and directed networks (Scotti *et al*. 2007). First, we calculated the direct plus indirect effects in unweighted networks. We defined *a*_*n,ij*_ as the effect of taxon *j* on taxon *i* when *i* can be reached from *j* in *n* steps. The simplest case of calculating *a*_*n,ij*_ is when *n *= 1:

(10) $$a_{1,ij}=\frac{b_{ij}}{\sum_{j=1}^{D}b_{ij}}$$

where *b*_*ij*_ is the element of the qualitative feeding matrix. Here, *a*_1,*i*,*j*_ = 1/*D*_*i*,*out*_, if species *j* is a consumer and *a*_1,*i*,*j*_ = 1/*D*_*i*,*in*_, if species *j* is a resource. *D*_*i*,*in*_ is the number of resources for taxon *i*, whereas *D*_*i*,*out*_ is the number of consumers for taxon *i*. Furthermore, we define the *n*‐step effect originating from species *i* by the following formula:

(11) $$\sigma_{n,i}=\sum_{j=1}^{S}a_{n,\;ji}$$

The direct and indirect effects originating from species *i* up to *n* steps are considered as:

(12) $$DI_{i}^{n}=\frac{\sum_{m=1}^{n}\sigma_{m,i}}{n}=\frac{\sum_{m=1}^{n}\sum_{j=1}^{S}a_{m,ji}}{n}$$

which represents the sum of effects originating from species *i* up to *n* steps averaged over by the maximum number of steps considered. Here, we considered a maximum of five‐step‐long indirect effects, i.e. *n* = 5. As the strength of indirect effects decreases dramatically with distance (Berlow *et al*. 2009; Borrett *et al*. 2010; Stouffer & Bascompte 2011), up to five steps are sufficient to get their precise value (Scotti *et al*. 2007; Borrett *et al*. 2010). For a weighted network, all the effects are defined in the same way as above except that the value of *b*_*ij*_ is the amount of biomass flowing from taxon *i* to taxon *j*.

We simulated taxon loss for each food web by sequentially removing taxa. We used the Adaptive Runge–Kutta method with adaptive step sizes to perform numerical simulations. In each simulation, the empirical biomass data were employed to give the initial biomass values. Thousand days were simulated first, to allow transient dynamics caused by initial effects to settle down and let the system reach steady state (Hudson & Reuman 2013). Then we started the sequential deletions, which can be seen as a stepwise process: 1000 days were simulated after each deletion, and secondary extinctions during this time were recorded. Before adding a new step, the deletion sequences were updated, as the extinctions in the former step would change network structure and carbon fluxes among the surviving taxa. During the simulation, a species was considered to be extinct if its biomass fell to < 10^−30^ g C m^−2^ (Berlow *et al*. 2009). We did not remove any detrital nodes in the extinction sequence (Staniczenko *et al*. 2010) to guarantee that energy cycling would occur during the simulations, which thus continued until only detrital nodes were left in the web. Note that an established food web may persist for a long period without autotrophs if detrital taxa have accumulated sufficient carbon storage to sustain detritus‐based organisms (see Appendix S2).

### Measures of stability

We employed two indices to characterise the stability of food webs: robustness (*R*_50_) and survival area (*SA*). Robustness was quantified as the proportion of species subjected to primary removals that resulted in 50% of total species loss, which is commonly used in such analyses (Dunne *et al*. 2002; Dunne & Williams 2009; Curtsdotter *et al*. 2011). A higher value of *R*_50_ means fewer secondary extinctions and thus higher stability. *SA* is the area under the curve resulting from plotting the number of surviving taxa, *N*_*P*,_ having occurred at a specific number of primary deletions, *p*. *SA* is calculated as

(13) $$SA=\frac{\sum_{p=1}^{S_{l}}N_{p}}{S_{l}^{2}}$$

where *S*_*l*_ is the number of living taxa in the original food web. The value of *SA* meets the term *SA *+ *EA *= 1, where *EA* means extinction area as used in prior studies (Allesina & Pascual 2009; Curtsdotter *et al*. 2011). Here, we chose *SA* rather than *EA* because it exhibits a positive relationship with stability, i.e. a higher value of *SA* indicates higher stability. All numerical simulations and calculations were carried out in Matlab (version 7.12.0).

### Statistical procedures

We conducted 1000 Monte‐Carlo simulations for each web and for each node‐ordering index, with four parameters (*h*,* k*_0_, *b* and *q*) varying randomly in each replicate (1000 replicates × 20 webs × 4 indices = 80 000 simulations). We separately compared the effects of the four indices (Max.D, Max.DI, Max.wD and Max.wDI) on *R*_50_ and *SA* using a linear mixed effects model (LME) with a maximum‐likelihood estimator (function ‘lme’ with ‘method = ML’ within the ‘nlme’ package in R 3.2.3). Food‐web identity was included in the model as a random factor to correct for differences between study systems. Post‐hoc comparisons were applied using the Tukey HSD test at α = 0.05 level of significance (function ‘glht’ within the ‘multcomp’ package). As robustness and connectance are logarithmically related (Dunne *et al*. 2002), we explored the relationship between stability and log transformations of the measures of complexity (i.e. *S*,* C* and *C*_*w*_), using the functions ‘lm’ and ‘cor’ in the ‘stats’ package.

## Results

**Figure 1:**
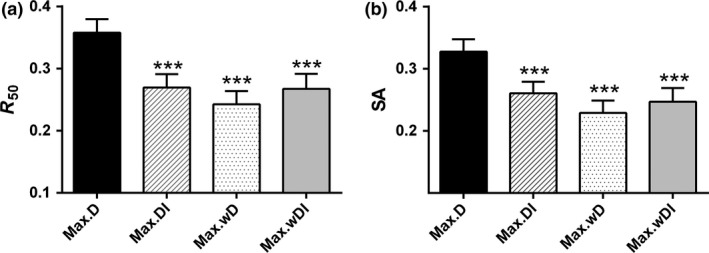
Stability of the 20 food webs to species loss in four deletion sequences (mean ± SEM). Here, stability is represented by (a) robustness, *R*_50_, the fraction of taxa that have to be removed to induce ≥ 50% total taxon loss, and (b) survival area, *SA*, the area under the curve resulting from plotting the number of surviving taxa. The stars directly above the error bars denote significant differences in stability between the focal deletion orders and the control order (Max.D): *** *P *< 0.001.

With the nonlinear functional response, the four deletion orders produced significantly different values of *R*_50_ (Fig. 1a, LME: *F*_3,57_ = 13.07, *P *< 0.001). Deletion orders Max.DI, Max.wD and Max.wDI had significantly lower values of *R*_50_ than order Max.D (Tukey test, see Table S1). There was no significant difference in *R*_50_ among the three deletion orders Max.DI, Max.wD and Max.wDI (Tukey test, Table S1). The four deletion orders also produced significantly different values of *SA* (Fig. 1b, LME: *F*_3,57_ = 12.072, *P *< 0.001). Again, the three new indices led to significantly lower values of *SA* than Max.D (Tukey test, Table S1). Using a linear functional response led to significantly lower stability than the nonlinear form (LME: *F*_1,79_ = 98.974, *P* < 0.001 for *R*_50_; *F*_1,79_ = 101.338, *P* < 0.001 for *SA*). The comparison of the four deletion orders produced similar results to the nonlinear functional response, however, with significantly different values of *R*_50_ (Fig. S2a, LME: *F*_3,57_ = 12.520, *P *< 0.001) and *SA* (Fig. S2b, LME: *F*_3,57_ = 25.048, *P *< 0.001), whereas Max.DI, Max.wD and Max.wDI led to significantly lower stability than Max.D (Tukey test, Table S1).

Further analyses showed that different values of the four free parameters (*h*,* k*_0_, *b* and *q*) in the nonlinear functional form did not alter our major conclusion for both *R*_50_ and *SA* (Figs S3–S6), i.e. deletions in Max.D led to significantly higher stability than the three new indices in all groups (Tukey test). With the linear functional response, the change in the only free parameter (*k*_0_) also did not alter this conclusion (Fig. S7).

**Figure 2:**
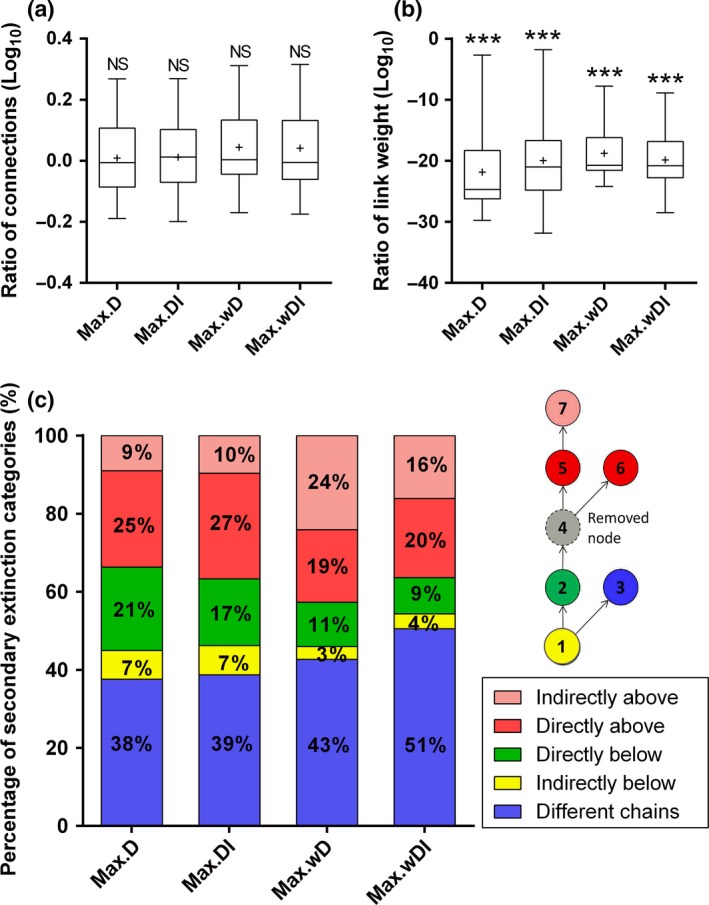
(a–b) Comparison of the types of nodes that went secondarily extinct with those surviving. The nodes going secondarily extinct were significantly different (denoted by stars) from the surviving nodes in (a) number of links or (b) link weight if the confidence intervals around the logarithm of the ratio between the value of the secondarily extinct nodes and the average value of all surviving nodes did not overlap with zero. (c) Trophic categories of nodes that caused the secondary extinctions. The percentage of secondary extinctions for each trophic category across the 1000 simulations of all 20 food webs is shown, along with an illustration of what each of the trophic categories imply.

There was no significant difference in the connectivity of nodes that went secondarily extinct compared with the average value of those that remained (Fig. 2a; *t*_19_ = 0.31, *P* = 0.762 for Max.D; *t*_19_ = 0.44, *P* = 0.667 for Max.DI; *t*_19_ = 1.65, *P* = 0.115 for Max.wD and *t*_19_ = 1.44, *P* = 0.167 for Max.wDI). There was a significant difference in the link weight of nodes that went secondarily extinct compared with the average value of those that remained (Fig. 2b; *t*_19_ = − 14.47, *P* < 0.001 for Max.D; *t*_19_ = − 12.66, *P* < 0.001 for Max.DI; *t*_19_ = − 19.03, *P* < 0.001 for Max.wD and *t*_19_ = − 18.40, *P* < 0.001 for Max.wDI). Most (54–71%) secondary extinctions were caused by indirect effects (the pink, yellow and purple groups in Fig. 2c). Bottom‐up cascades, which are the only cause of collateral losses in the topological approach, accounted for about 40% of secondary extinctions (the red and pink groups in Fig. 2c).

**Figure 3:**
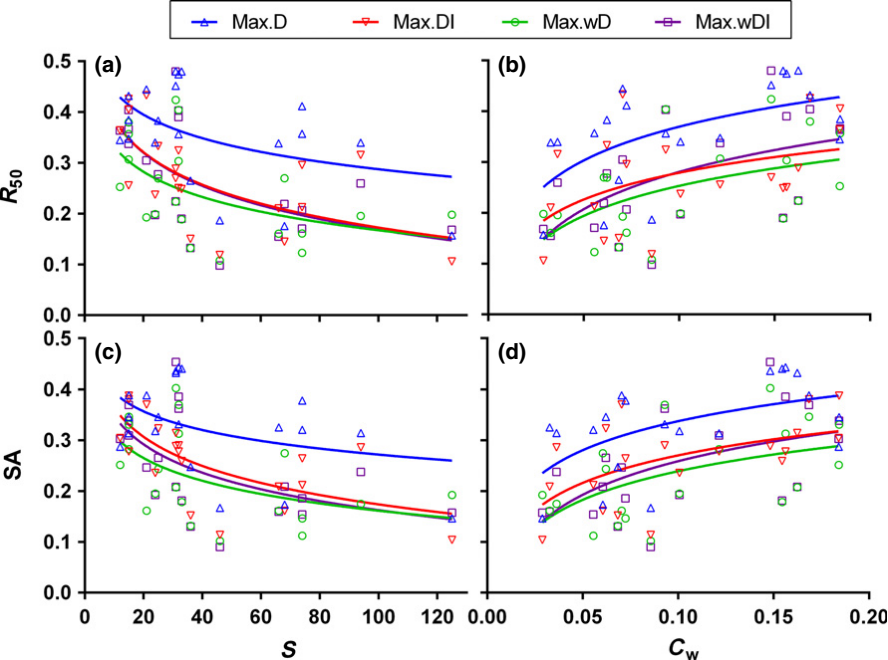
Stability in nonlinear functional response simulations indicated by robustness, *R*_50_ (top panels), and survival area, *SA* (bottom panels), as a function of the taxon richness, *S* (left panels), and weighted directed connectance, *C*_*w*_ (right panels), of each food web. Logarithmic fits to the four datasets are shown, with different colours and markers indicating different deletion orders.

Across all 20 food webs, the stability indicated by *R*_50_ and *SA* under the four deletion orders with the nonlinear functional response varied significantly with *S* and *C*_*w*_, but rarely with *C* (Table 3 and Fig. 3). More specifically, *R*_50_ and *SA* decreased with increasing Log *S* (except *SA* in Max.D) and with decreasing Log *C*_*w*_ (Table 3). There was no significant effect of the different deletion orders on the slopes of Log *R*_50_ vs. Log *S* (Two‐way anova:* F*_3,72_ = 0.31, *P* = 0.821) and Log *C*_*w*_ (*F*_3,72_ = 0.07, *P* = 0.977). Similarly, there was no significant effect of the different deletion orders on the slopes of Log *SA* vs. Log *S* (*F*_3,72_ = 0.34, *P* = 0.795) and Log *C*_*w*_ (*F*_3,72_ = 0.09, *P* = 0.968). The same patterns emerged from the dynamical models based on a linear functional response (Fig. S8). Although food‐web stability increased with decreasing food‐web size, we found that the inevitable decrease in the size of a food web during the deletion process seldom affected our conclusion (see Appendix S3).

**Table 3:** Stability of food webs under four different species deletion sequences as a function of three measures of food‐web complexity

Stability	Deletion sequences	*Log S*			*Log C*			*Log C*_*w*_		
		Slope	*P*	*r* ^2^	Slope	*P*	*r* ^2^	Slope	*P*	*r* ^2^
*R*_50_	Max.D	**− 0.067**	**0.043**	**0.21**	**0.201**	**< 0.001**	**0.69**	**0.096**	**0.008**	**0.33**
	Max.DI	**− 0.092**	**0.002**	**0.41**	0.081	0.141	0.12	**0.076**	**0.040**	**0.21**
	Max.wD	**− 0.072**	**0.022**	**0.26**	0.065	0.238	0.08	**0.085**	**0.016**	**0.28**
	Max.wDI	**− 0.094**	**0.007**	**0.34**	0.112	0.065	0.18	**0.106**	**0.008**	**0.33**
*SA*	Max.D	**− **0.053	0.083	0.16	**0.186**	**< 0.001**	**0.71**	**0.082**	**0.013**	**0.30**
	Max.DI	**− 0.083**	**0.001**	**0.45**	**0.096**	**0.035**	**0.22**	**0.078**	**0.010**	**0.31**
	Max.wD	**− 0.064**	**0.030**	**0.23**	0.065	0.209	0.09	**0.081**	**0.015**	**0.29**
	Max.wDI	**− 0.081**	**0.012**	**0.30**	0.107	0.051	0.20	**0.095**	**0.009**	**0.33**

Linear regressions of robustness, *R*_50_ (the fraction of species that have to be removed to induce ≥ 50% total species loss), and survival area, *SA* (the area under the curve resulting from plotting the number of survival taxa), of 20 food webs to species loss following four deletion sequences as a function of the logarithm of taxon richness (*S*), binary directed connectance (*C*) and weighted directed connectance (*C*_*w*_). Significant results (*P* < 0.05) are shown in bold.

## Discussion

In the last few decades, the influence of random loss of nodes (‘error’) and selective loss of the most‐connected nodes (‘attack’) has been investigated in many real‐world networks, e.g. the Internet (Albert *et al*. 2000). All of these networks exhibit high fragility against the removal of the most‐connected nodes (i.e. ‘hubs’), which in an ecological context suggests these nodes would represent keystone species that play an important role in maintaining biodiversity (Dunne *et al*. 2002; Memmott *et al*. 2004; Dunne & Williams 2009). However, this conclusion is drawn based on topological approaches that always underestimate the amount of secondary extinctions (Curtsdotter *et al*. 2011). Recent studies using a static structural approach have found that node ordering would be altered when considering link weight or indirect effects (Jordán *et al*. 2006; Scotti *et al*. 2007; Bauer *et al*. 2010), suggesting the possibility of more useful centrality indices. Using a food‐web dynamical model derived from empirical energy budget data, we found that network stability was significantly lower when deletions were ordered by indirect effects, link weight or both, compared with the ordering by unweighted degree centrality (see Fig. 1). Furthermore, poorly connected nodes faced the same extinction risk as highly connected nodes, whereas nodes with low link weight were more likely to go extinct secondarily (see Fig. 2a,b), indicating the failure of degree centrality and the importance of weighted indices. Over 50% of secondarily extinct nodes were not directly connected to the removed nodes (see Fig. 2c), emphasising the need to consider indirect effects. These findings suggest that indices considering link weight and indirect effects are better descriptors of centrality in food webs than the traditional binary, direct‐link measure. Moreover, our findings were robust to different forms of the functional response and different values of the hill exponent, carrying capacity coefficient, half‐saturation coefficient and predator interference coefficient, suggesting a high level of generality.

The uneven distribution of interaction strengths in food webs (O'Gorman *et al*. 2010) provides a cautionary note when interpreting results derived from analyses of simple binary networks (Banašek‐Richter *et al*. 2009). It is generally assumed that specialised species tend to have strong connections, whereas generalised species have weak interactions (Wootton & Emmerson 2005) and hence weaker net effects (Montoya *et al*. 2009; O'Gorman *et al*. 2010) and different contributions to network structure and stability relative to poorly connected species. We found that species removals ordered by link weight led to a > 30% reduction in network stability relative to direct unweighted orderings. This conclusion is important because it suggests that studies focused solely on direct, unweighted indices for identifying key species in food webs may have severely overestimated the relative importance of degree centrality and, although successfully identifying topologically important nodes, they may fail to detect functionally important ones. Notice that considering weights did not add anything to the conclusion as long as indirect effects were considered, which might be caused by the significant correlation between Max.DI and Max.wDI in 18 of the 20 food webs (Spearman rank correlation = 0.557 ± 0.037; mean ± SEM).

Most empirical studies only contain qualitative food‐web data due to logistical constraints in collecting quantitative information on link weights, although the situation is improving (Ings *et al*. 2009). Our results show that food‐web stability is significantly lower after removals ordered by both unweighted direct and indirect effects than by direct effects alone. Thus, in the absence of quantitative data, an understanding of indirect effects will give a more realistic view of species importance than in a network constructed solely from direct‐link information. This is perhaps unsurprising, given that indirect effects can often be stronger than direct effects in food webs (Werner & Peacor 2003; Salas & Borrett 2011). Trophic cascades and apparent competition are the best known examples of indirect effects (Montoya *et al*. 2009). Indirect effects have also been regarded as important drivers of secondary extinctions in a recent empirical study (Sanders *et al*. 2013). In our study, indirect effects accounted for over 50% of the secondary extinctions (Fig. 2c). This implies that not only neighbouring links but also neighbours of neighbours need to be considered to better understand how species losses propagate. For example, in the well‐studied Chesapeake ecosystem, zooplankton have the most direct links to other taxa, whereas bacteria in sediment POC have the strongest direct plus indirect and weighted effects (see Table S2 for the other food webs).

The hypothesis that diversity may give rise to ecosystem stability has led to more than half a century of heated debate in ecology (May 1972; Tilman *et al*. 2006). Many experiments have shown that higher diversity is associated with a reduction in temporal variability (i.e. increased temporal stability; Tilman *et al*. 2006; Cardinale *et al*. 2009), but the opposite may be true when considering other measures of stability (Donohue *et al*. 2013). Our study demonstrated a negative relationship between species richness and the robustness of food webs to secondary extinction under all four of the deletion scenarios, suggesting that even species‐rich ecosystems can be vulnerable to cascading extinctions. A possible explanation for this is density compensation, i.e. population densities decrease with increasing species richness because of the increased intensity of interspecific competition (Borrvall & Ebenman 2008; Kaneryd *et al*. 2012). Our data showed a strong negative correlation between average biomass densities and taxon richness (Fig. S9, Pearson correlation coefficient *r* = − 0.95, *P* < 0.001), which supported this hypothesis. As a consequence, species are more easily excluded because they are closer to their extinction threshold, a finding consistent with previous dynamical analyses (Borrvall & Ebenman 2008; Kaneryd *et al*. 2012).

We found a strong positive relationship between weighted directed connectance, *C*_*w*_, and food‐web stability, but a surprisingly weak relationship with binary directed connectance, *C*. This stands in contrast to earlier topological analyses (Dunne *et al*. 2002; Dunne & Williams 2009) conducted on binary networks of direct links. When topological approaches are used, highly connected communities are robust to species loss because species with many binary links are unlikely to become isolated and thus go extinct. Using a dynamical approach, however, highly connected nodes face a similar extinction risk as poorly connected nodes (Fig. 2a). In this case, the density of weighted carbon flows plays an increasingly important role, where the loss of a particularly strong link may result in a node receiving insufficient energy to persist in the network, even if it retains several weak connections to other nodes. Therefore, the nodes with lower link weight would have a significantly higher risk of going secondarily extinct (Fig. 2b). This is also consistent with recent findings that increasing the energy threshold for consumer secondary extinction would nullify the previously positive relationship between robustness and binary directed connectance (Thierry *et al*. 2011; Bellingeri & Bodini 2013). The dynamical approach, through the weighting of links refines our understanding of the factors affecting network stability in ways that topological analyses cannot do because they assign equal importance to all connections in the network (Eklöf & Ebenman 2006; Curtsdotter *et al*. 2011).

Moreover, in the topological approach, nodes are considered to be extinct only when they lose all their resources, so all secondary extinctions emerge from bottom‐up cascades (Eklöf & Ebenman 2006). In dynamical approaches, however, a node cannot persist if it receives insufficient energy, even though it still has resources. This is in agreement with a recent study (Bellingeri & Bodini 2013), which investigated the effects of the thresholds of minimum energy requirement for species survival on the robustness of food webs. Top‐down effects and other effects mediated by exploitative and apparent competition can also play an important role (Elmhagen & Rushton 2007; Säterberg *et al*. 2013). In our study, bottom‐up effects only accounted for about half of all the secondary extinctions (Fig. 2c), highlighting the potential for dynamical analyses to identify a significant proportion of secondary extinctions that would otherwise be missed with topological approaches.

As we enter the age of the sixth mass extinction (Barnosky *et al*. 2011), we need efficient indices to quantify the relative importance of species to develop new management policies for prioritising key populations to be conserved (Waldron *et al*. 2013). Our study contributes towards potential solutions and may help ecologists to outline a better conservation policy based on the functional importance of species, rather than qualitative metrics such as rarity or ‘hubs’. By quantifying link weights (or in the absence of quantitative link data, by considering indirect effects) we can improve the accuracy of keystone species identification (Jordán *et al*. 2008). The extent to which our methods help in detecting more accurate indices remains to be seen, but we posit that it will improve the designs of subsequent experiments or dynamical simulation studies.

## Data Accessibility

All data used in this study are available in the ‘enaR’ package (Borrett & Lau 2014) in R (https://cran.r-project.org/web/packages/enaR/index.html).

## Authorship

HZ and GW were responsible for research design. LZ drafted the main text and prepared the figures. SRB processed the data. LZ, HZ and JCM developed the modelling framework. LZ and WT performed numerical simulation. EOG, LZ and AM analysed the results. All authors were involved in discussions and editing.
