# Supplementary material for: Weighting and indirect effects identify keystone species in food webs
Source: Ecol Lett. 2016 Jun 27;19(9):1032–40. doi: 10.1111/ele.12638 (PMC5008267; doi:10.1111/ele.12638)
Supplement: Supplementary file 1 [file ELE-19-1032-s001.docx]

**SUPPORTING INFORMATION**

**Weighting and indirect effects identify keystone species in food webs**

**Lei Zhao^a, b^, Huayong Zhang^* a^, Eoin J. O'Gorman^b^, Wang Tian^a^, Athen Ma^c^,**

**John C. Moore^d, e^, Stuart R. Borrett^f, g^, and Guy Woodward^b^**

*^a^ Research Center for Engineering Ecology and Nonlinear Science, North China Electric Power University, Beijing, 102206, China*

*^b^ Department of Life Sciences, Imperial College London, Silwood Park Campus, Buckhurst Road, Ascot, Berkshire, SL5 7PY, UK*

*^c^ School of Electronic Engineering and Computer Science, Queen Mary, University of London, Mile End Road, London, E1 4NS, UK*

*^d^ Department of Ecosystem Science and Sustainability, Colorado State University, Fort Collins, CO 80523, USA*

*^e^ Natural Resource Ecology Laboratory, Colorado State University, Fort Collins, CO 80523, USA*

*^f^ Department of Biology and Marine Biology, University of North Carolina Wilmington,, Wilmington, NC 28403, USA*

*^g^* *Duke Network Analysis Center, Duke University, Durham, NC 27708, USA*

*Corresponding author: Huayong Zhang, E-mail: rceens@ncepu.edu.cn

**Supplementary Methods and Analyses**

**Appendix S1.** Calculating weighted directed connectance.

**Appendix S2.** “Green” webs and “brown” webs.

**Appendix S3.** Effects of food web shrinkage during the node deletion process.

**Supplementary Tables**

**Table S1.** Multiple comparisons of robustness (*R_50_*) or survival area (*SA*) under four different deletion sequences, with different functional responses.

**Table S2.** Taxa with the three highest values for the four deletion orders in each food web.

**Supplemental Figures**

**Figure S1.** Scheme of dynamical model construction based on carbon flux.

**Figure S2.** Stability, represented by (a) robustness, *R_50_*, and (b) survival area, *SA*, to species loss in four deletion sequences with a linear functional response.

**Figure S3.** Stability, represented by (a) robustness, *R_50_*, and (b) survival area, *SA*, to species loss in four deletion sequences in five groups of hill exponent, *h*, with a nonlinear functional response.

**Figure S4.** Stability, represented by (a) robustness, *R_50_*, and (b) survival area, *SA*, to species loss in four deletion sequences in five groups of carrying capacity coefficient, *k_0_*, with a nonlinear functional response.

**Figure S5.** Stability, represented by (a) robustness, *R_50_*, and (b) survival area, *SA*, to species loss in four deletion sequences in five groups of half-saturation coefficient, *b*, with a nonlinear functional response.

**Figure S6.** Stability, represented by (a) robustness, *R_50_*, and (b) survival area, *SA*, to species loss in four deletion sequences in five groups of predator interference coefficient, *q*, with a nonlinear functional response.

**Figure S7.** Stability, represented by (a) robustness, *R_50_*, and (b) survival area, *SA*, to species loss in four deletion sequences in five groups of carrying capacity coefficient, *k_0_*, with a linear functional response.

**Figure S8.** Stability in linear functional response simulations indicated by robustness, *R_50_*, and survival area, *SA*, as a function of the taxon richness *S* and weighted connectance, *C_w_* of each web.

**Figure S9.** Linear regression of average logarithmic biomass and taxon richness of 20 food webs.

**Figure S10.** Comparison of energy channels from producers and detritus.

**Figure S11.** The ratios of detritus storage (DS) to daily net primary production (NPP) and daily respiration of the detritus-based food web (R).

**Figure S12.** Stability, represented by survival area (*SA*), to species loss in four deletion sequences, when the criterion to end the simulation once all producers were extinct was applied.

**Figure S13.** The ratio of the fractions of taxa that have to be removed to cause a food web to lose 0-50% (R_1_) and 50-100% (R_2_) of its living taxa for the four deletion sequences.

**Figure S14.** The proportion of relative food web sizes (the ratio of the size after deletion to the starting size) in ten size classes for the four deletion sequences.

**Figure S15.** Pielou’s evenness of the distribution of relative food web sizes in ten size classes for the four deletion sequences and uniform distribution.

**Supplementary References**

**Appendix S1. Calculating weighted directed connectance**

Here we summarized the method for calculating weighted directed connectance *C_w_* after Banašek-Richter *et al.*(2009): a food web with *S* species can be represented by an *S*-by-*S* quantitative food-web matrix ***b*** = [*b_ij_*]; the value of the element *b_ij_* means the amount of biomass passing from taxon *i* to taxon *j* per unit area and time; for taxon *k*, we can measure the diversity of the biomass coming from its resources (*H_R,k_*) and of that going to its consumers (*H_C,k_*), i.e. the taxon-specific Shannon indices of inflows and outflows:

 (1)

 (2)

Here means the sum of column *k*, while represents the sum of row *k*. The effective number of resources (*N_R,k_*) and consumers (*N_C,k_*) are:


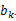

$N_{R,k}=\exp(H_{R,k})$ (3)


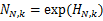

$N_{C,k}=\exp(H_{C,k})$ (4)


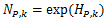


The weighted link density (*LD_w_*) is:

 (5)

where is the total sum of the matrix. Lastly, the weighted directed connectance (*C_w_*) can be obtained as following:

$C_{w}=\frac{LD_{w}}{S}$ (6)

**Appendix S2. “Green” webs and “brown” webs**

During the sequential deletions, it was possible for all producers to go extinct. One logical way to deal with this issue may be to discard any simulations where this occurred, to avoid including food webs with no autotrophic energy in the analyses. However, since food webs can be divided into producer-based (“green”) and detritus-based (“brown”) sub-webs (Butler *et al.* 2008; Rooney *et al.* 2008), the brown food webs may still function and persist for a long time if the detrital taxa have sufficient carbon storage. In the main text, we let the dynamical model determine the loss of nodes based on the energy budget, rather than making the judgment artificially (based on presence and absence of both green and brown sub-webs). In this section, we explore the relative sizes of the two sub webs and the detritus storage for each ecosystem. Furthermore, we tested whether our conclusions are altered if we add the new criterion that simulations are discarded when all producers go extinct.

First, we explored the role of detritus in the 20 food webs. A method proposed by Rooney *et al.* (2008) was used to calculate the proportion of carbon derived from producers and detritus:

$\%BR_{C}=\sum_{1}^{n} P_{C}\times\%BR_{R}$ (7)


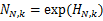


where %BR_C_ means the proportion of carbon derived from basal resources, *n* is the number of resources consumed by the consumer, *P_C_* is the proportion of the consumer diet accounted for by a resource, and %BR_R_ means the proportion of carbon derived from the basal resource in the resource being consumed. Using this method, we calculated the proportion of “green” energy and “brown” energy for each node in each food web (see Fig. S10a for an example). If a node obtained more than half of its energy from producers, we counted it in the green sub-web; if it derived more energy from detritus, we counted it in the brown sub-web. We found that, on average, 43% of nodes were detritus-based, and 30% of the 20 food webs were dominated by brown webs (see Fig. S10b).

The average carbon storage of detritus in the 20 ecosystems was approximately equal to the accumulated net primary production after 186 days (Fig. S11). The average carbon storage of detritus supported 603 days of brown web respiration, assuming that all the taxa in the brown webs had constant respiration. In reality, the population biomass and respiration of consumers would decrease as energy from producers is cut off. The exhaustion of detritus would also decrease the total biomass and thus the total energy demand of the brown webs, which would extend the persistent period of brown webs even further.

Considering the large size of detritus-based food webs and the large storage of detritus, it would be reasonable to let the dynamical model determine the loss of nodes based on the energy budget. However, it is still useful to explore whether our conclusions are altered if we add the criterion that the simulations are discarded if all producers go extinct. In this case *R_50_*, the fraction of taxa that have to be removed in order to induce ≥50% total taxon loss, may not be reached in some food webs because all producers go extinct earlier than half of the total taxa. Thus we only used survival area, *SA*, to test if our conclusions held. Based on our results, the three new indices led to significantly lower stability than Max.D (see Fig. S12, *z* = -2.831, *P* = 0.028 for Max.DI; *z* = -6.098, *P* < 0.001 for Max.wD; *z* = -3.771, *P* = 0.001 for Max.wDI), so our major conclusion was not altered.

**Appendix S3 Effects of food web shrinkage during the node deletion process**

During the sequential deletion process, the size of the food webs kept decreasing as primary and secondary extinctions occurred. In this section, we test whether this web shrinkage would affect the comparison among deletion sequences. This question can be separated into two parts: (1) whether stability changed as the food webs shrank in size during species deletion; and (2) whether large and small webs were equally represented in the four deletion sequences.

First, we tested if stability changed as the food webs shrank in size during node deletion. Let R_1_ and R_2_ be the fractions of taxa that have to be removed to cause a food web to lose 0-50% and 50-100% of its living taxa, respectively. If stability increases as food webs shrink, we would expect R_1_/R_2_ < 1, i.e. fewer primary deletions are needed to lose the first 50% of living taxa compared to the last 50%. We found that this ratio was not significantly different from 1 for the deletion orders Max.D (*t*-test: *t_19_* = -0.568, *P* = 0.577) and Max.wDI (*t_19_* = -1.066, *P* = 0.300), but it was significantly less than 1 for Max.DI (*t_19_* = -2.505, *P* = 0.011) and Max.wD (*t_19_* = -3.246, *P* = 0.002). The average values of R_1_/R_2_ were all close to 1, however (see Fig. S13; 0.973 ± 0.048 (mean ± SEM), 0.787 ± 0.085, 0.800 ± 0.062, and 0.912 ± 0.083 for Max.D, Max.DI, Max.wD, and Max.wDI, respectively), indicating that stability did not change much as food webs shrank during species deletion.

Second, we tested if small and large webs were equally represented for each deletion sequence. We binned the relative size of the food webs (the ratio of the size after deletion to the starting size) into ten size classes and calculated the proportion for each size class (see Fig. S14). We then calculated the Pielou’s evenness of the relative size distribution for each deletion sequence for each web. To test if the relative size was evenly distributed, for each web we conducted 1,000 Monte Carlo simulations. In each simulation, *N* random numbers from U[0, 1] were generated. *N* is the mean number of deletions for the four deletion sequences, representing the number of web sizes in deletion sequences. The proportions of these numbers in the ten bins were counted and Pielou’s evenness was calculated for each simulation for each web. The mean evenness of the 1,000 simulations was used to compare with the evenness of the four deletion sequences, using a linear mixed effects model (LME) with a maximum-likelihood estimator (function ‘lme’ with ‘method = ML’ within the ‘nlme’ package in R 3.2.3). Food web identity was included in the model as a random factor. Post-hoc comparisons were applied using the Tukey HSD test at α = 0.05 level of significance (function ‘glht’ within the ‘multcomp’ package).

The evenness of Max.D (*z* = -4.960, *P* < 0.001) and Max.DI (*z* = --3.661, *P* = 0.003) were significantly less even than the uniform distribution, while Max.wD (*z* = -1.185, *P* = 1) and Max.wDI (*z* = -1.859, *P* = 0.631) were not significantly different from the uniform distribution (Fig. S15). Among the four deletion sequences, only two pairs showed significant differences in evenness (*z* = 3.775, *P* = 0.002 for Max.D *vs* Max.wD; *z* = 3.102, *P* = 0.019 for Max.D *vs* Max.wDI). The average evenness of the four deletion sequences (0.228 ± 0.014, 0.239 ± 0.010, 0.260 ± 0.013, and 0.254 ± 0.014 respectively) were very close to the uniform distributions (0.270 ± 0.013), suggesting that food web sizes were evenly distributed and there was negligible bias towards small or large webs.

**Table S1.** Multiple comparisons of robustness (*R_50_*) or survival area (*SA*) under four different deletion sequences, with different functional responses. Significant results (*P* < 0.05) are shown in bold.

| Functional response | Stability | Deletion sequences | Max.D | Max.DI | Max.wD | Max.wDI |
| --- | --- | --- | --- | --- | --- | --- |
| Nonlinear | *R_50_* | Max.D | － | **-4.469** | **-5.836** | **-4.575** |
|  |  | Max.DI |  | － | -1.367 | -0.106 |
|  |  | Max.wD |  |  | － | 1.261 |
|  |  | Max.wDI |  |  |  | － |
|  | *SA* | Max.D | － | **-3.823** | **-5.631** | **-4.602** |
|  |  | Max.DI |  | － | -1.808 | -0.779 |
|  |  | Max.wD |  |  | － | 1.029 |
|  |  | Max.wDI |  |  |  | － |
| Linear | *R_50_* | Max.D | － | **-3.247** | **-5.728** | **-4.733** |
|  |  | Max.DI |  | － | -2.481 | -1.487 |
|  |  | Max.wD |  |  | － | 0.995 |
|  |  | Max.wDI |  |  |  | － |
|  | *SA* | Max.D | － | **-4.856** | **-8.274** | **-6.377** |
|  |  | Max.DI |  | － | **-3.417** | -1.521 |
|  |  | Max.wD |  |  | － | 1.896 |
|  |  | Max.wDI |  |  |  | － |

**Table S2.** Taxa with the three highest values for the four deletion orders in each food web.

| **Food web** | **Max.D** | **Max.DI** | **Max.wD** | **Max.wDI** |
| --- | --- | --- | --- | --- |
| **Bothnian Bay** | Mesozooplakton | Macrofauna | Bacteria | Meiofauna |
|  | Dem. Fish | Meiofauna | Microzooplakton | Bacteria |
|  | Macrofauna | Mesozooplakton | Mesozooplakton | Microzooplakton |
| **Baltic Sea** | Deposit Feeders | Deposit Feeders | Mesozooplakton | Mesozooplakton |
|  | Benthis Invertebrate Carnivores | Meiofauna | Pelagic Production | Deposit Feeders |
|  | Mesozooplakton | Mesozooplakton | Microzooplankton | Pelagic Production |
| **Ems Estuary** | Benthis Invertebrate Carnivores | Benthis Invertebrate Carnivores | Meiofauna | Microzooplankton |
|  | Microzooplankton | Meiofauna | Microzooplankton | Meiofauna |
|  | Mesozooplankton | Microzooplankton | Benthis Producers | Pelagic Producers |
| **Swartkops** | Planktivorous Fish | Microzooplankton | Benthic Suspention Feeders | Benthic Suspention Feeders |
|  | Microzooplankton | Planktivorous Fish | Benthis Producers | Benthis Producers |
|  | Mesozooplankton | Mesozooplankton | Meiofauna | Microzooplankton |
| **Crystal River** | Benthis Invertebrate Carnivores | Benthis Invertebrate Carnivores | Macrophytes | Benthis Invertebrate Carnivores |
|  | Gulf Killifish | Zooplankton | Benthis Invertebrate Carnivores | Macrophytes |
|  | Pinfish | Pinfish | Microphytes | Microphytes |
| **Benguela** | Hake | Microplankton | Bacteria | Microplankton |
|  | Carnivorous Fish | Phytoplankton | Phytoplankton | Bacteria |
|  | Macrozooplankton | Mesozooplankton | Mesozooplankton | Phytoplankton |
| **Neuse Estuary** | Meiobenthos | Meiobenthos | Free living bacteria | Sediment bacteria |
|  | Demersal fish | Sediment bacteria | Sediment bacteria | Zooplankton |
|  | Pelagic-demersal fish | Dep. feed. polychaetes | Zooplankton | Free living bacteria |
| **Georges Bank** | Macrobenthos- crustace | Macrobenthos- other | Phytoplankton- Primary | Phytoplankton- Primary |
|  | Macrobenthos- other | Macrobenthos- crustace | Small copepods | Macrobenthos- other |
|  | Demersals- piscivores | Sharks- pelagics | Bacteria | Bacteria |
| **Gulf of Maine** | Macrobenthos- crustacea | Macrobenthos- crustacea | Large Copepods | Large Copepods |
|  | Macrobenthos- other | Macrobenthos- other | Phytoplankton- Primary | Phytoplankton- Primary |
|  | Demersals- omnivores | Demersals- omnivores | Small copepods | Bacteria |
| **Narragansett** | Ben Macrofauna | Mesozooplankton | SedPOC Bacteria | SedPOC Bacteria |
|  | Shrimp(Pal+Crg) | Phytoplankton | Pelag Bacteria | Pelag Bacteria |
|  | Mesozooplankton | Shrimp(Pal+Crg) | Mesozooplankton | Phytoplankton |
| **Atlantic Bight** | Macrobenthos- crustace | Macrobenthos- crustace | Bacteria | Macrobenthos- other |
|  | Macrobenthos- other | Macrobenthos- other | Phytoplankton- Primary | Bacteria |
|  | Small Pelagics- commer | Sharks- coastal | Macrobenthos- other | Phytoplankton- Primary |
| **New England** | Macrobenthos- crustace | Macrobenthos- crustace | Phytoplankton- Primary | Phytoplankton- Primary |
|  | Macrobenthos- other | Macrobenthos- other | Bacteria | Bacteria |
|  | Demersals- omnivores | Sharks- pelagics | Small copepods | Small copepods |
| **Chesapeake** | Zooplankton | Bacteria in sediment POC | Bacteria in sediment POC | Bacteria in sediment POC |
|  | Bacteria in suspended POC | Blue Crab | Phytoplankton | Phytoplankton |
|  | Bay Anchovy | Zooplankton | Free Bacteria | Other Polychaetes |
| **St. Marks** | Predatory polycht | Benthic bact | Benthic bact | Benthic bact |
|  | Omnivorous crabs | Benthic algae | Meiofauna | Meiofauna |
|  | Benthic algae | Microfauna | Benthic algae | Benthic algae |
| **Graminoids** | Mesoinverts | Mesoinverts | Periphyton | Living Sediments |
|  | Other Macroinverts | Other Macroinverts | Floating Veg. | Periphyton |
|  | Mink | Mink | Macrophytes | Floating Veg. |
| **Cypress** | Terrst. I | Terrst. I | Living SED | Living SED |
|  | Snakes | Aquatic I | Cypress L | Periphyton |
|  | Fish PC | Fish PC | Periphyton | Fish HO |
| **Lake Oneida** | Insects | Cormorants | Diatoms | Amphipods |
|  | Leeches | Burbot | Blue-green Algae | Diatoms |
|  | Walleye Age 0 | Northern Pike | Epiphytes | Daphnia pulicaria |
| **Bay of Quinte** | Walleye Age-0 | Longnose Gar | Diatoms | Diatoms |
|  | Insects | Northern Pike | Blue-green Algae | Oligochaetes |
|  | Yellow Perch Age 1+ | Walleye Age-0 | Eubosmina coregoni | Blue-green Algae |
| **Mangroves** | SNKS | OTH. PP | BACT.SED. | BACT.SED. |
|  | TURT | INSCT | LEAF | OTH. PP |
|  | COCO | BACT.SED. | OTH. PP | LEAF |
| **Florida Bay** | Predatory Shrimp | Raptors | Water Flagellates | Water Flagellates |
|  | Pink Shrimp | Crocodiles | Thalassia | Benthic Flagellates |
|  | Herbivorous Shrimp | Benthic Phytoplankton | Benthic Flagellates | Meiofauna |


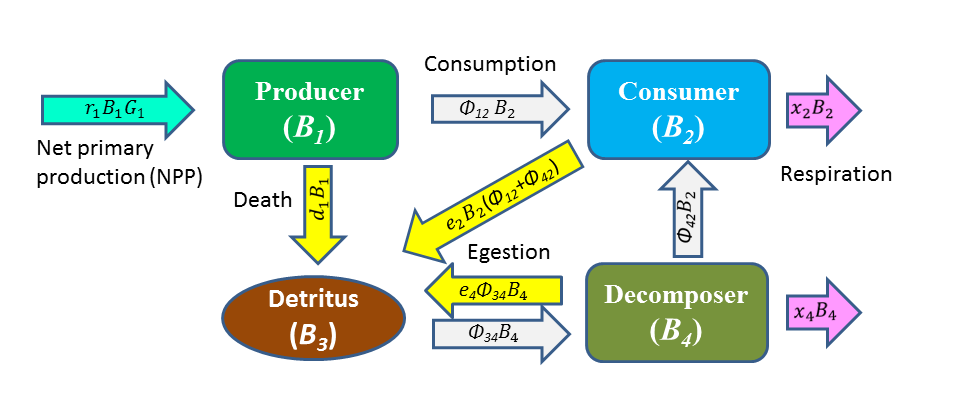


**Figure S1.** Scheme of dynamical model construction based on carbon flux. The food web is simplified into four compartments for clear demonstration: producers, consumers, decomposers, and detritus. The imports and exports via animal migration and water flows are considered to be in balance and not to influence the food web dynamics.


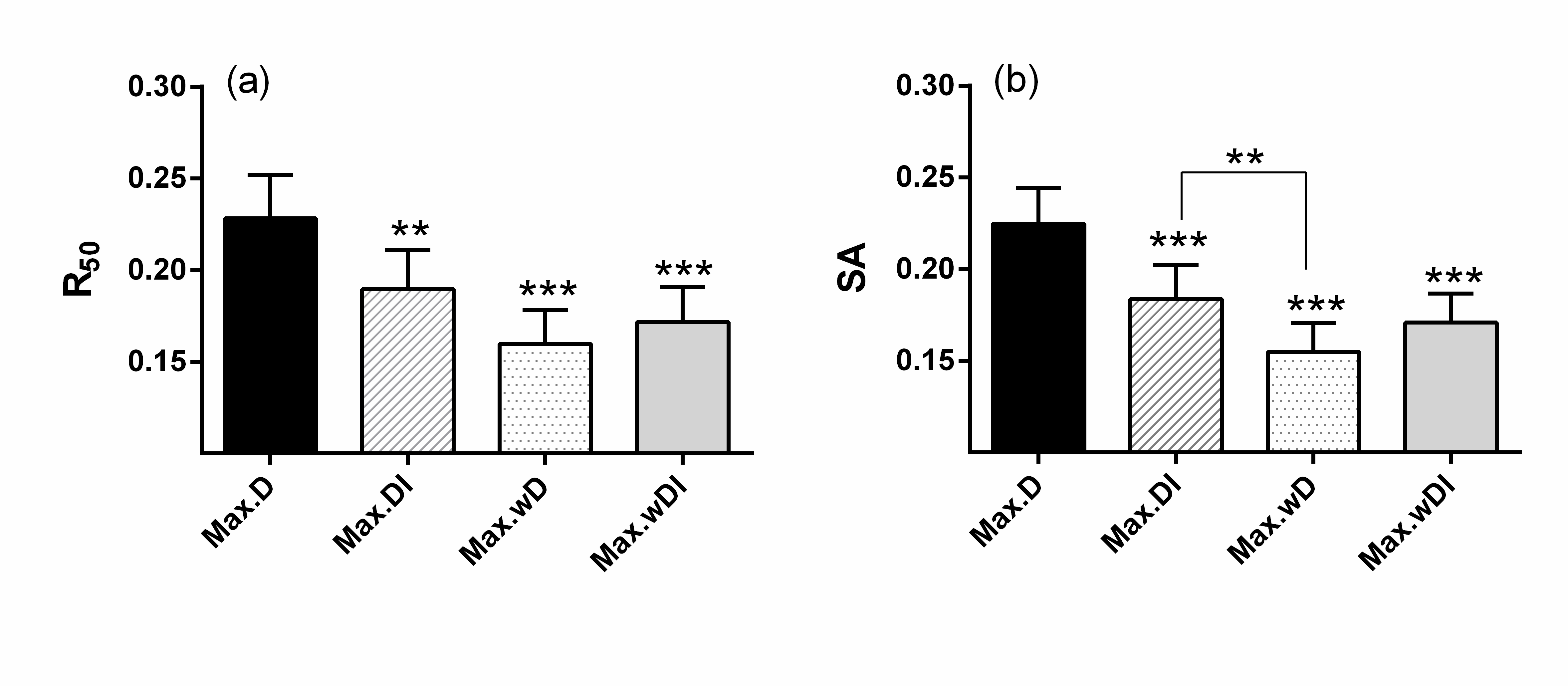


**Figure S2.** Stability, represented by (a) robustness, *R_50_*, and (b) survival area, *SA*, to species loss in four deletion sequences (mean ± SEM) with a linear functional response. The stars directly above the error bars denote significant differences in stability between the focal deletion orders and the control order (Max.D), detected using LME and Tukey post hoc test at 0.05 level of significance. Significant differences in the stability of deletion orders Max.DI, Max.wD, and Max.wDI are indicated by stars on lines connecting the compared indices: ********p* < 0.001; *******p* < 0.01; * *p* < 0.05; and NS, not significant.


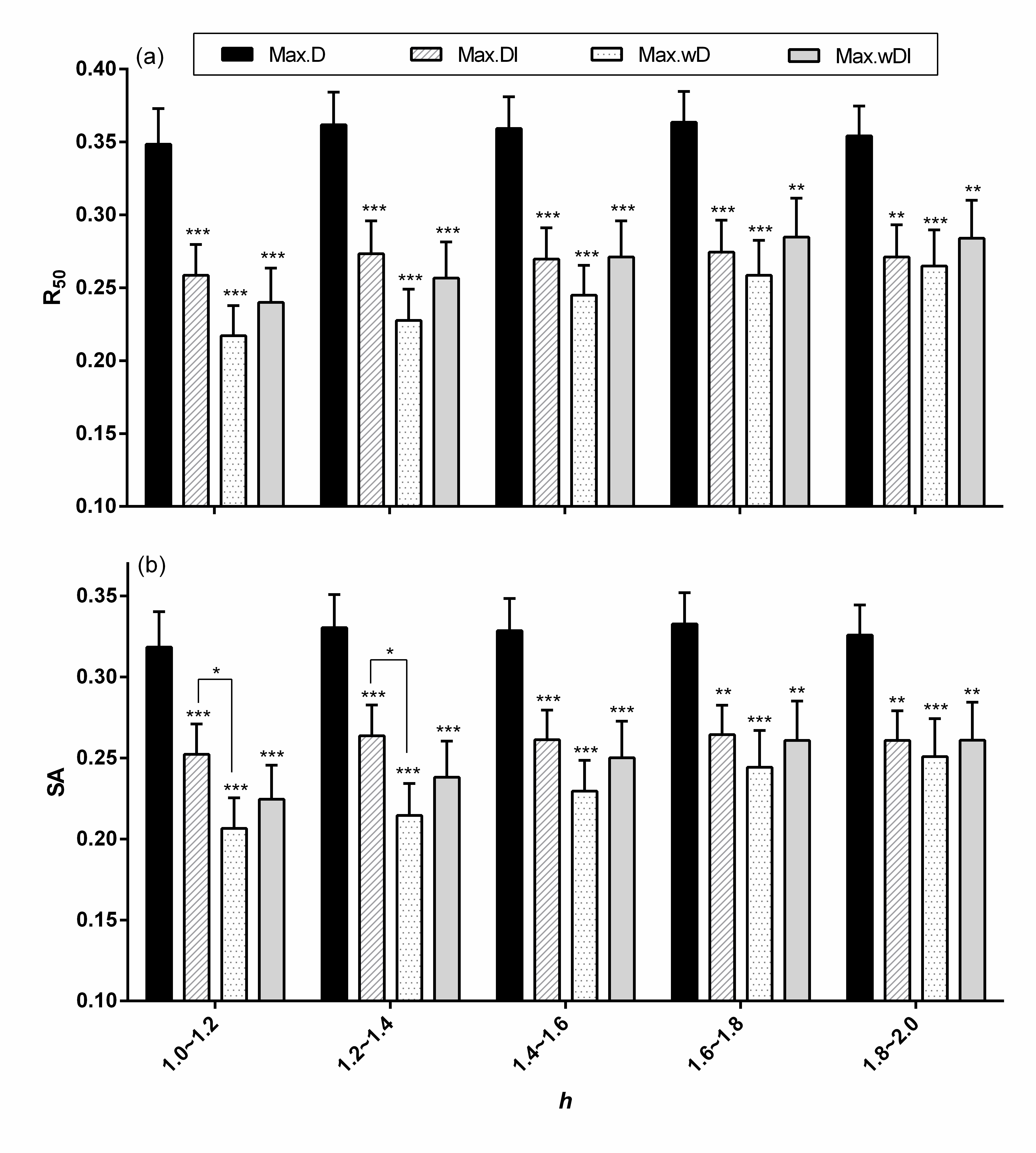


**Figure S3.** Stability, represented by (a) robustness, *R_50_*, and (b) survival area, *SA*, to species loss in four deletion sequences (mean ± SEM) in five groups of hill exponent, *h*, with a nonlinear functional response. The stars directly above the error bars denote significant differences in stability between the focal deletion orders and the control order (Max.D), detected using LME and Tukey post hoc test at 0.05 level of significance. Significant differences in the stability of deletion orders Max.DI, Max.wD, and Max.wDI are indicated by stars on lines connecting the compared indices: ********p* < 0.001; *******p* < 0.01; and * *p* < 0.05.


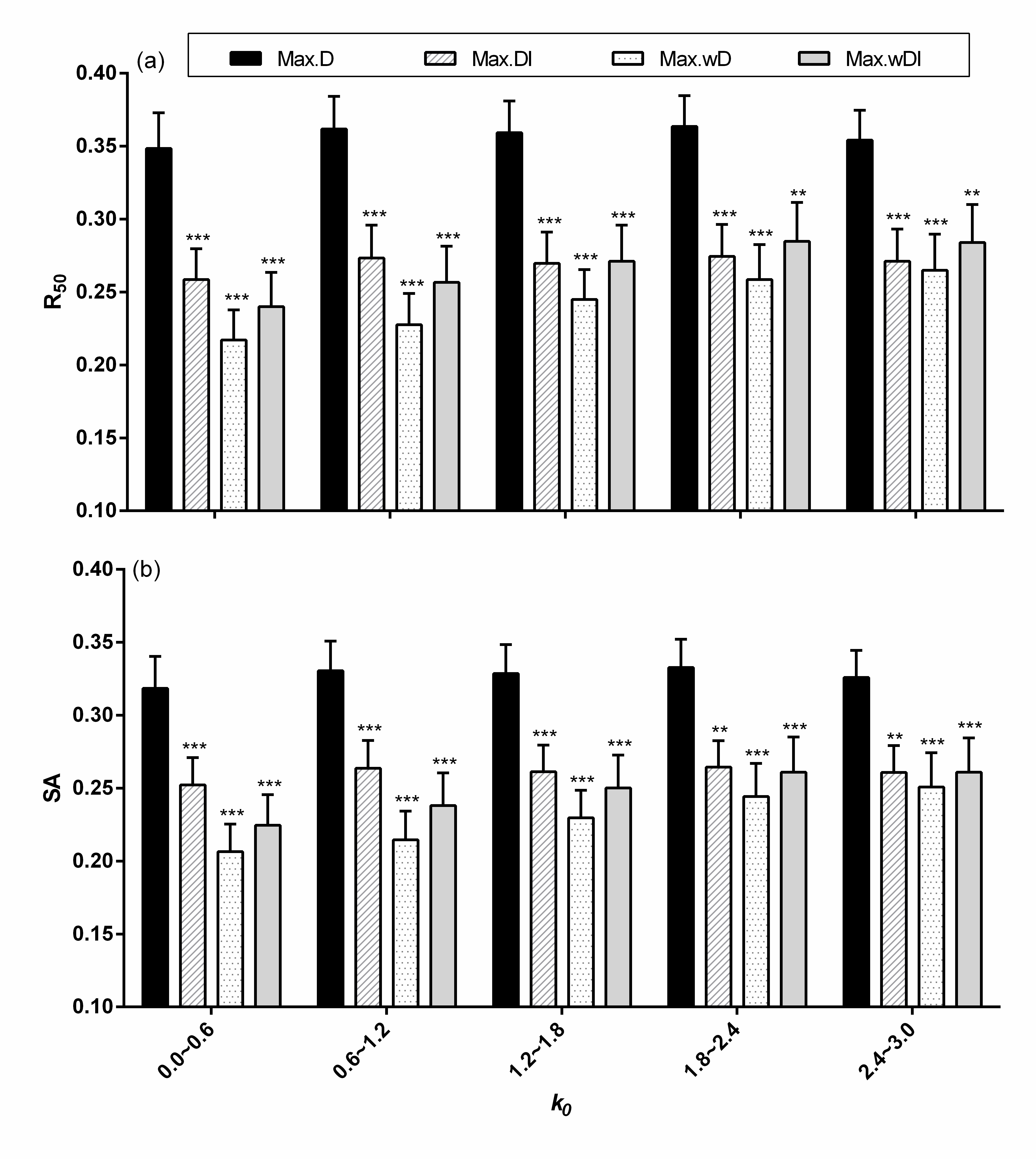


**Figure S4.** Stability, represented by (a) robustness, *R_50_*, and (b) survival area, *SA*, to species loss in four deletion sequences (mean ± SEM) in five groups of carrying capacity coefficient, *k_0_*, with a nonlinear functional response. The stars denote significant differences in stability between the focal deletion orders and the control order (Max.D), detected using LME and Tukey post hoc test at 0.05 level of significance: ********p* < 0.001; *******p* < 0.01; * *p* < 0.05; and NS, not significant.


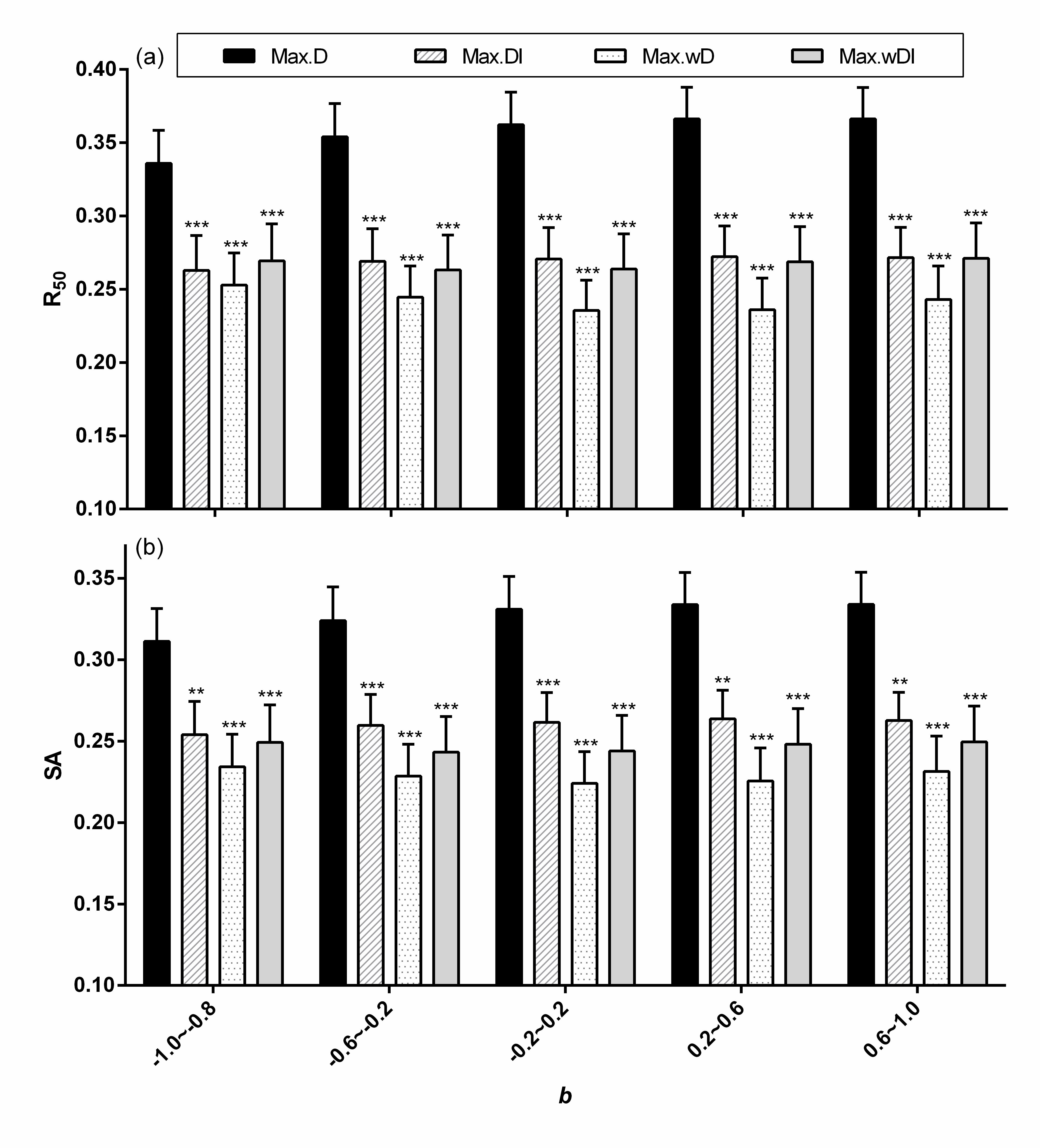


**Figure S5.** Stability, represented by (a) robustness, *R_50_*, and (b) survival area, *SA*, to species loss in four deletion sequences (mean ± SEM) in five groups of half-saturation coefficient, *b*, with a nonlinear functional response. The stars denote significant differences in stability between the focal deletion orders and the control order (Max.D), detected using LME and Tukey post hoc test at 0.05 level of significance: ********p* < 0.001; *******p* < 0.01; * *p* < 0.05; and NS, not significant.


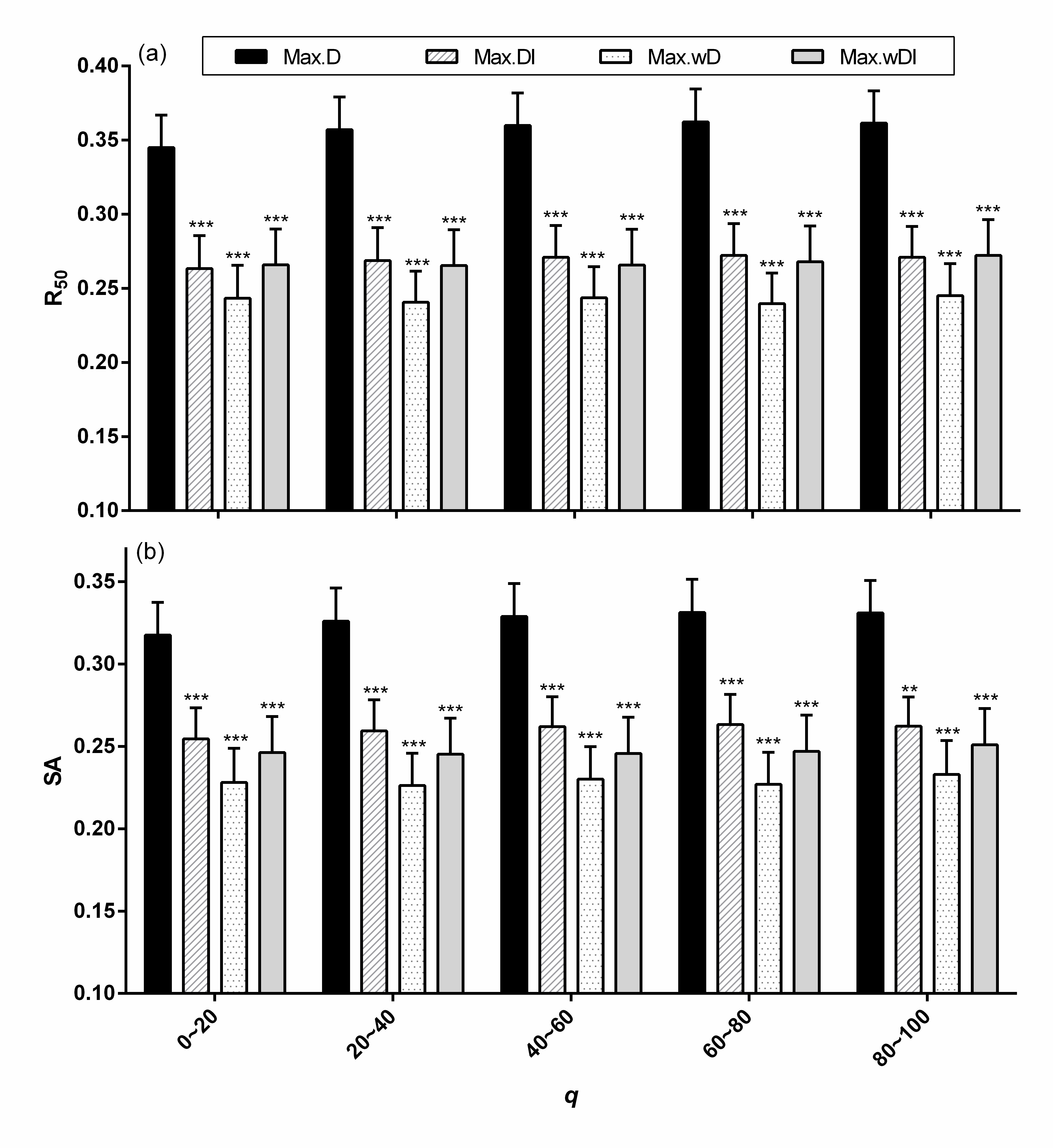


**Figure S6.** Stability, represented by (a) robustness, *R_50_*, and (b) survival area, *SA*, to species loss in four deletion sequences (mean ± SEM) in five groups of predator interference coefficient, *q*, with a nonlinear functional response. The stars denote significant differences in stability between the focal deletion orders and the control order (Max.D), detected using LME and Tukey post hoc test at 0.05 level of significance: ********p* < 0.001; *******p* < 0.01; * *p* < 0.05; and NS, not significant.


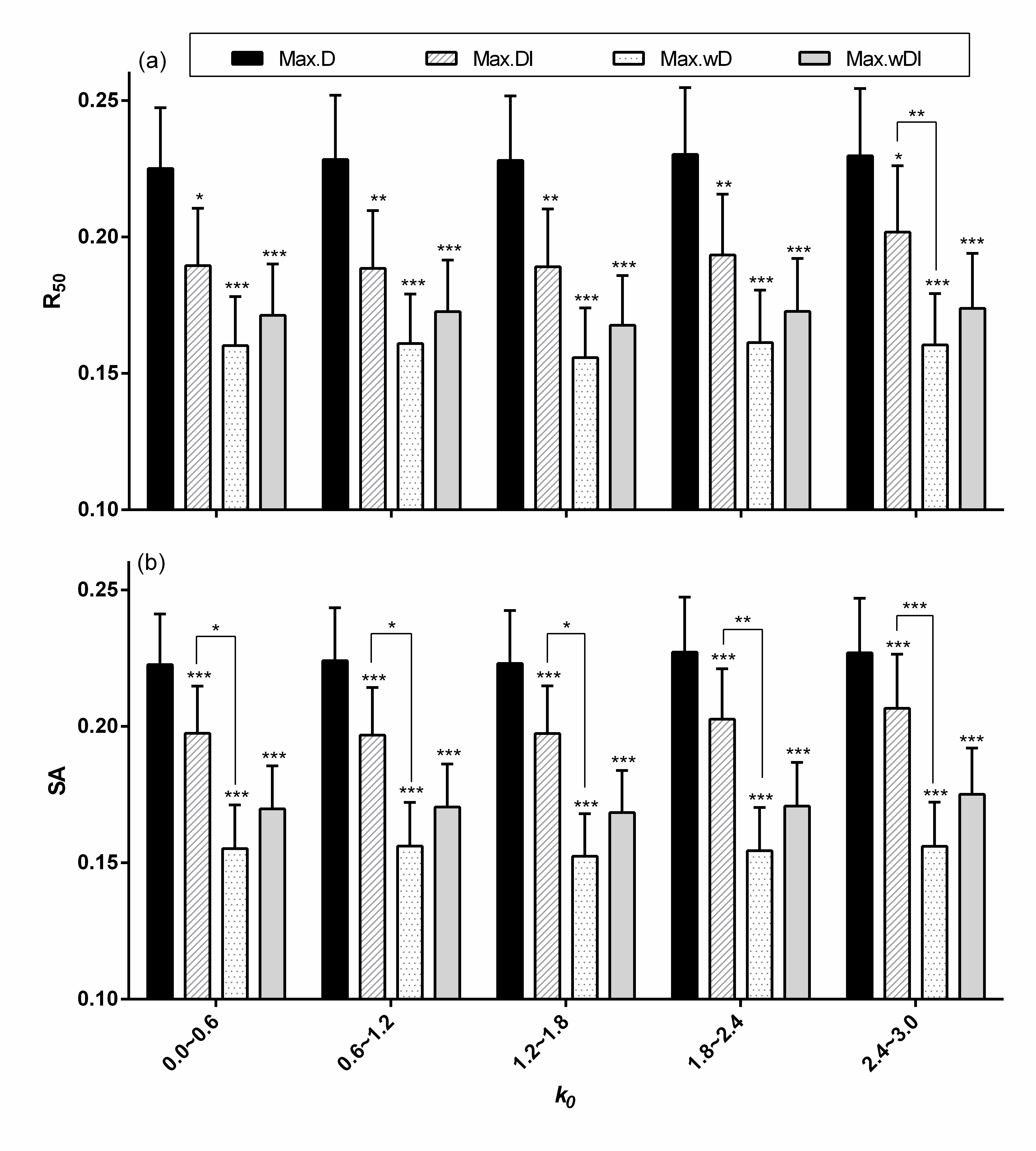


**Figure S7.** Stability, represented by (a) robustness, *R_50_*, and (b) survival area, *SA*, to species loss in four deletion sequences (mean ± SEM) in five groups of carrying capacity coefficient, *k_0_*, with a linear functional response. The stars denote significant differences in stability between the focal deletion orders and the control order (Max.D), detected using LME and Tukey post hoc test at 0.05 level of significance: ********p* < 0.001; *******p* < 0.01; * *p* < 0.05; and NS, not significant.


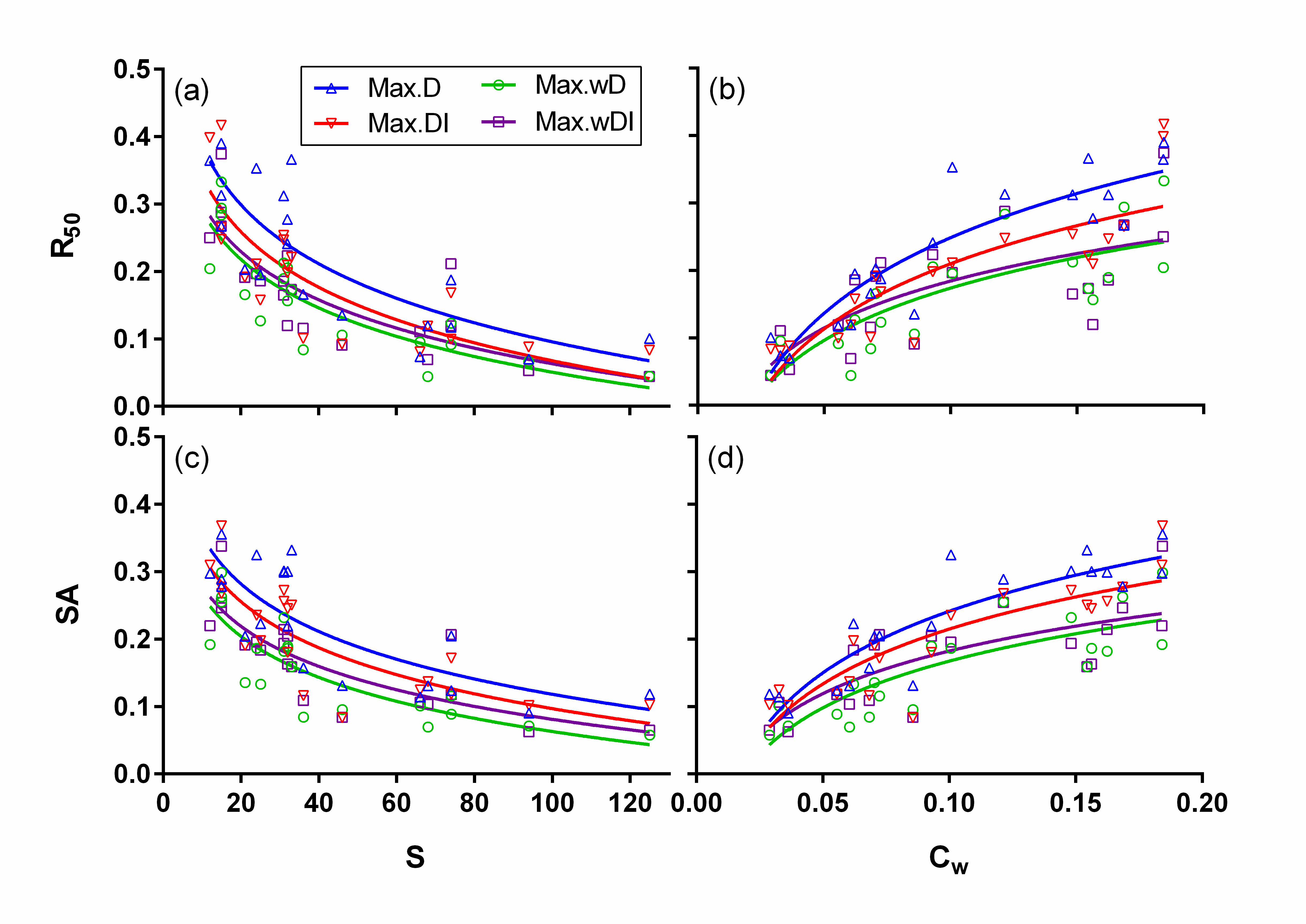


**Figure S8.** Stability in linear functional response simulations indicated by robustness, *R_50_* (top panels), and survival area, *SA* (bottom panels), as a function of the taxon richness *S* (left panels) and weighted connectance, *C_w_* (right panels) of each web. Logarithmic fits to the four data sets are shown, with different colours and markers indicating different deletion orders. The maximum possible *y*-value is 0.50.


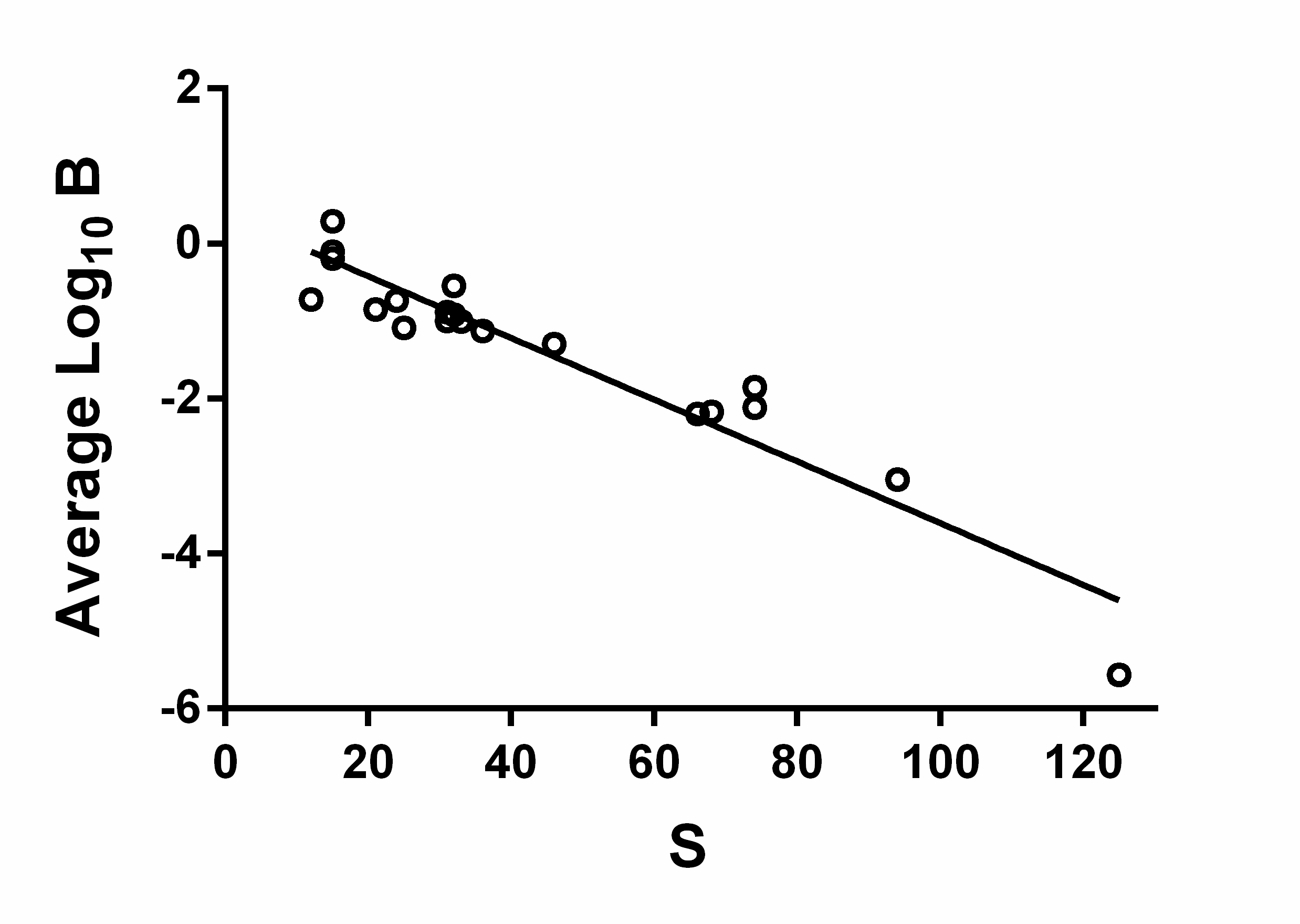


**Figure S9.** Linear regression of average logarithmic biomass and taxon richness of 20 food webs. The equation for the fit is Y = -0.040X + 0.377 (*F*_1,18_ = 165.8, *P* < 0.001, *r*^2^ = 0.90).


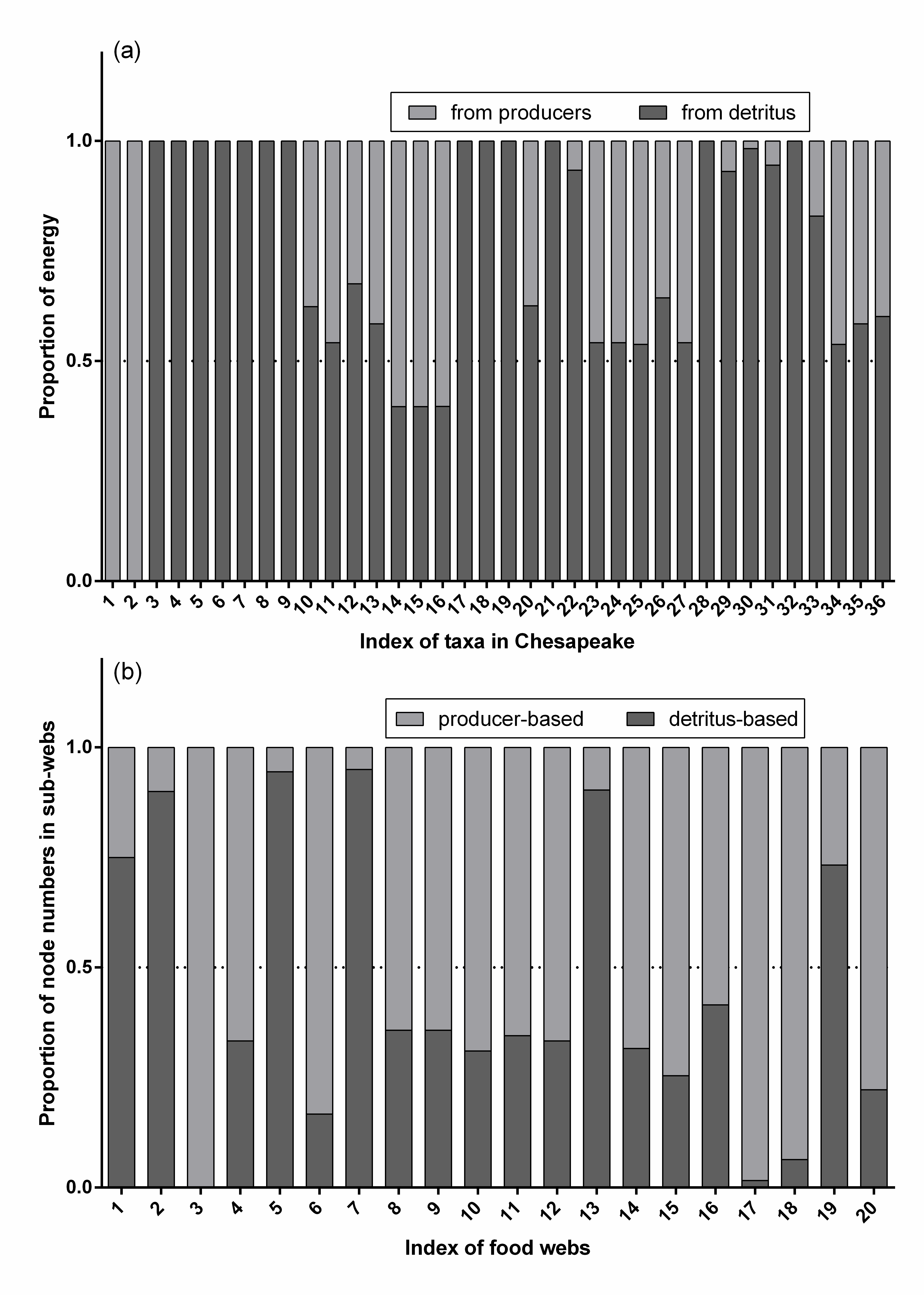


**Figure S10.** Comparison of energy channels from producers and detritus. (a) Proportion of energy derived from producers (grey) and detritus (dark) for each taxon in Chesapeake ecosystem. (b) Proportion of node numbers in producer-based (grey) sub-web and detritus-based (dark) sub-web for each ecosystem.


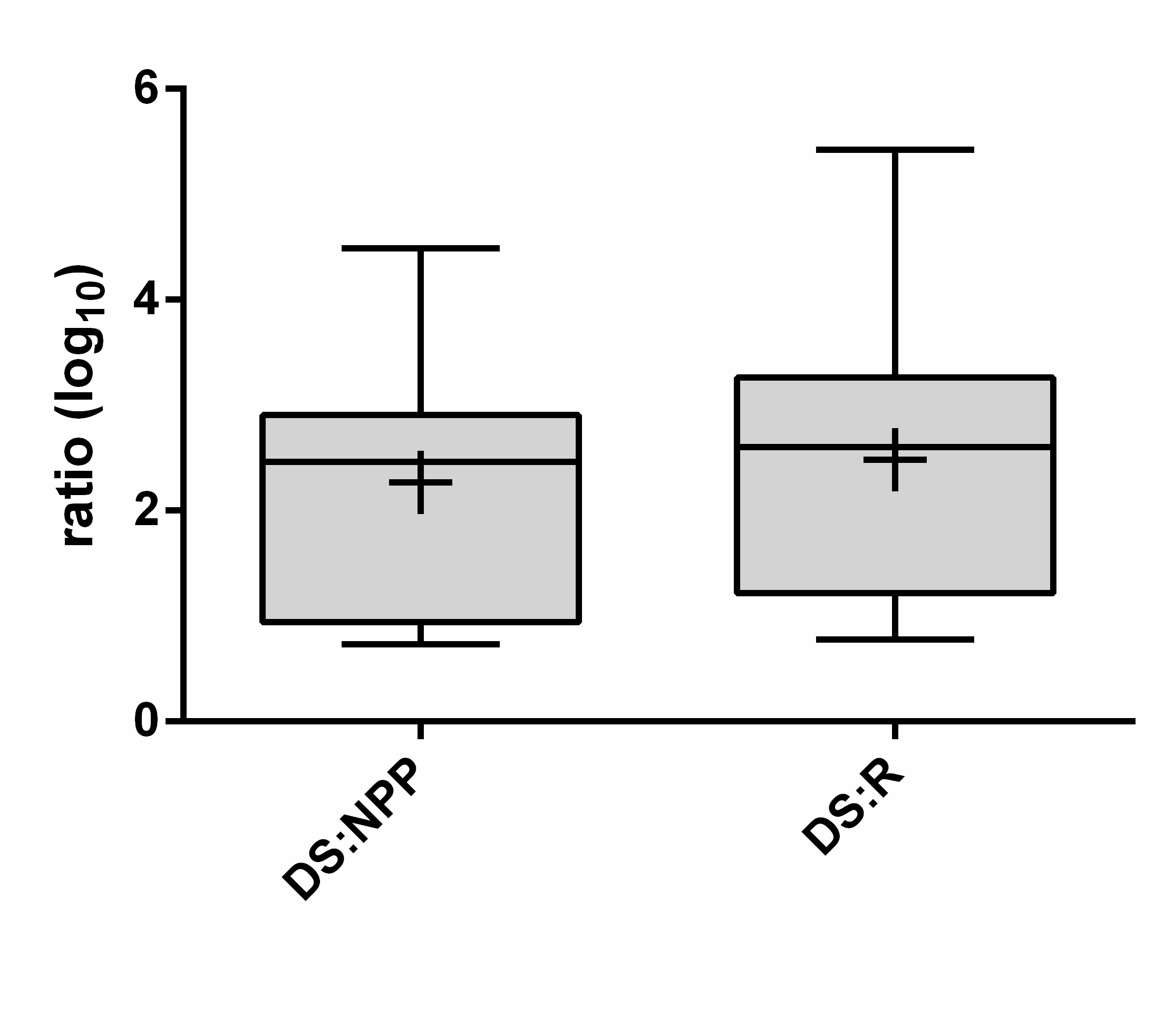


**Figure S11.** The ratios of detritus storage (DS) to daily net primary production (NPP) and daily respiration of the detritus-based food web (R).


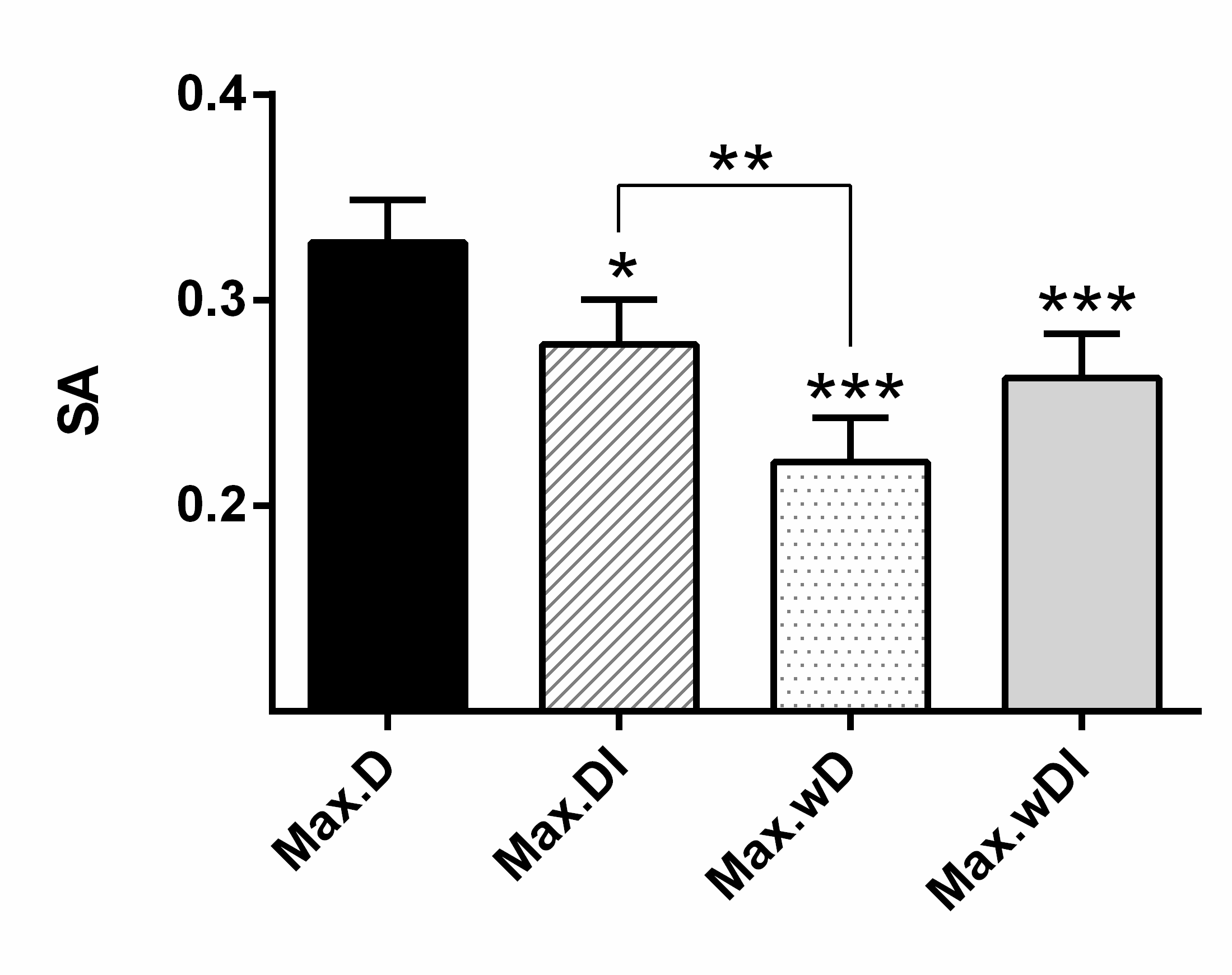


**Figure S12.** Stability, represented by survival area (*SA*), to species loss in four deletion sequences (mean ± SEM), when the criterion to end the simulation once all producers were extinct was applied. The stars directly above the error bars denote significant differences in stability between the focal deletion orders and the control order (Max.D), detected using LME and Tukey post hoc test at 0.05 level of significance. Significant differences in the *SA* of deletion orders Max.DI, Max.wD, and Max.wDI are indicated by stars on lines connecting the compared indices: ********p* < 0.001; *******p* < 0.01; and * *p* < 0.05.


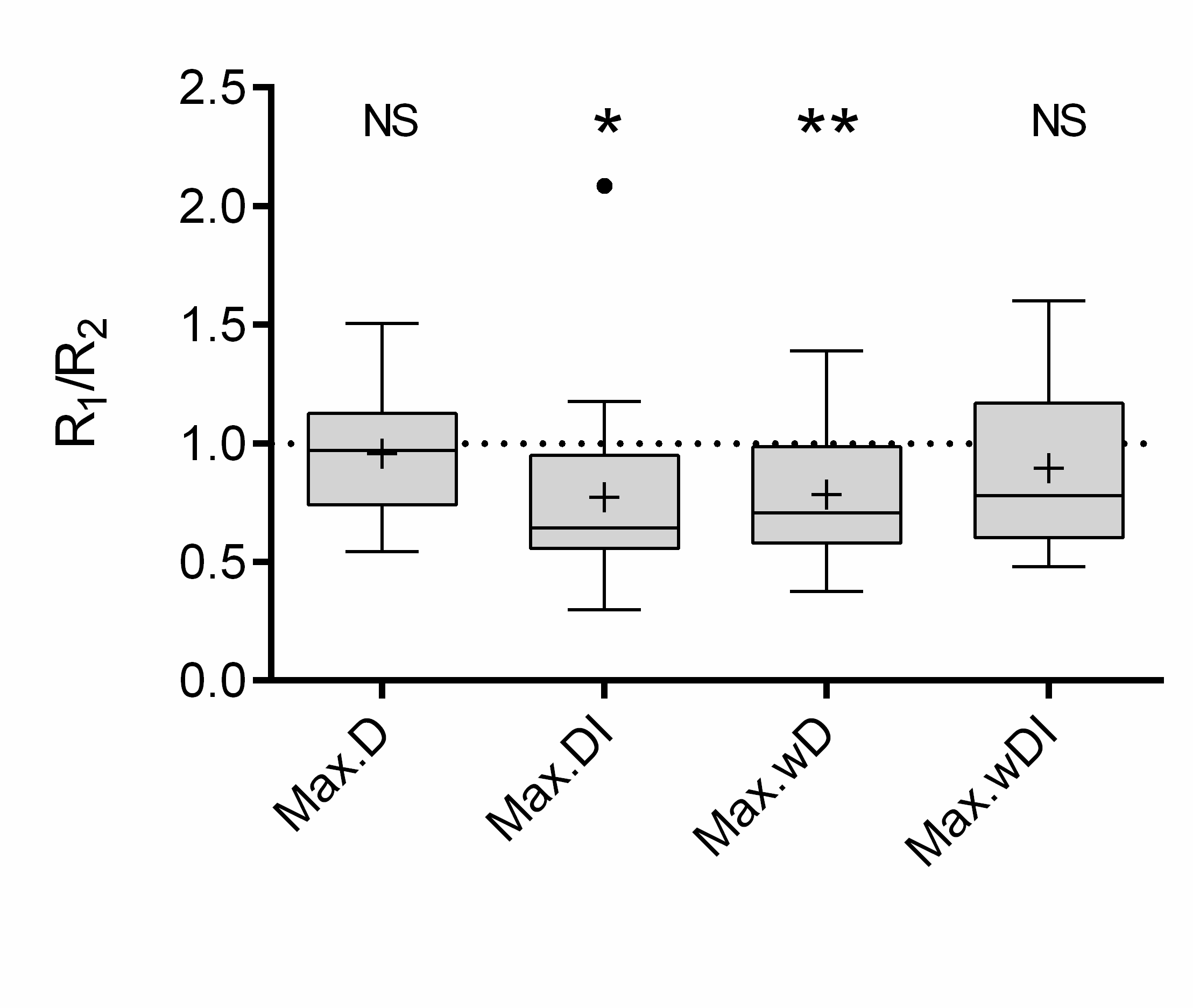


**Figure S13.** The ratio of the fractions of taxa that have to be removed to cause a food web to lose 0-50% (R_1_) and 50-100% (R_2_) of its living taxa for the four deletion sequences. Significant difference of R_1_/R_2_ from 1 was detected using t-test: ********p* < 0.001; *******p* < 0.01; and * *p* < 0.05.


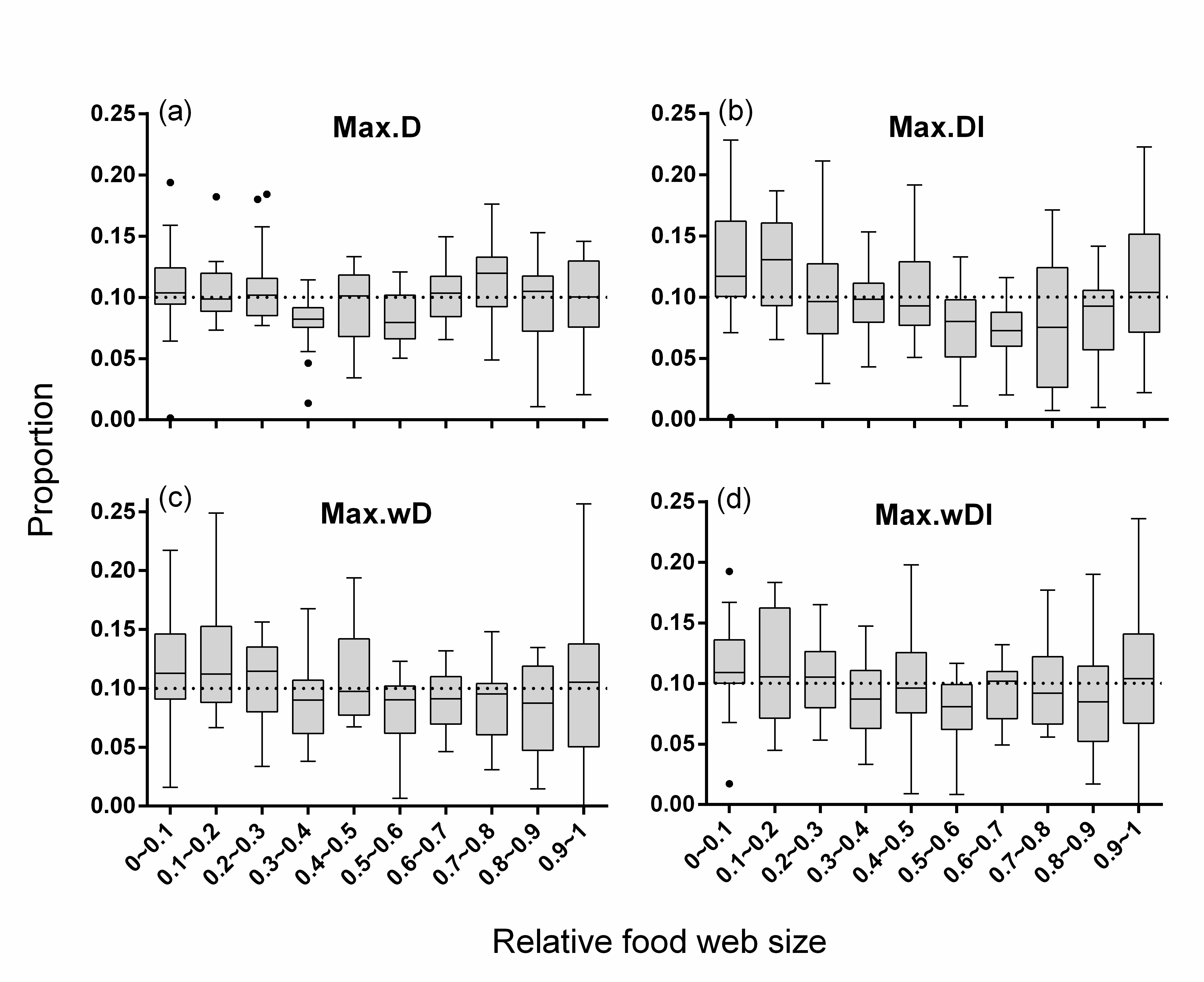


**Figure S14.** The proportion of relative food web sizes (the ratio of the size after deletion to the starting size) in ten size classes for the four deletion sequences.


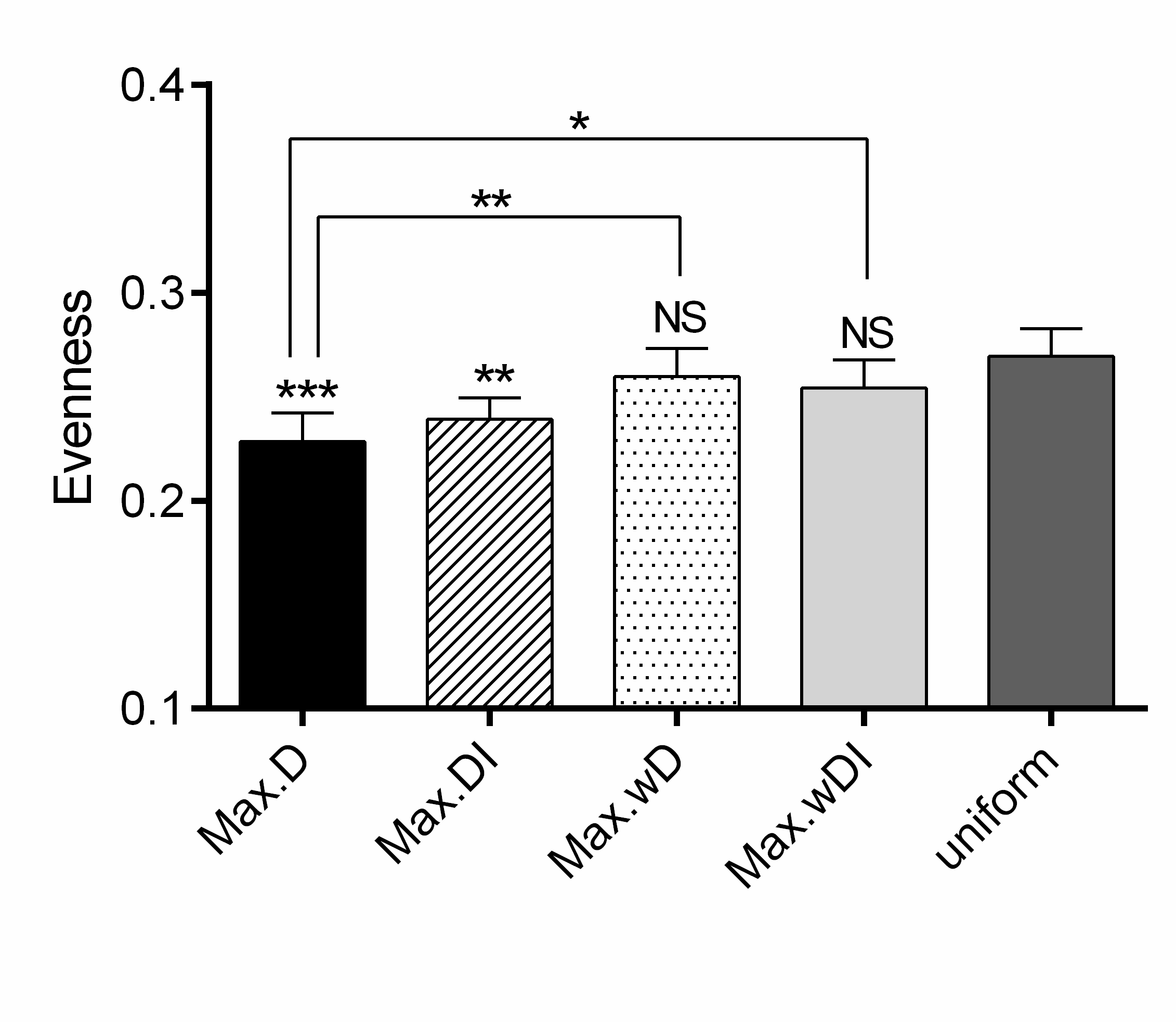


**Figure S15.** Pielou’s evenness of the distribution of relative food web sizes in ten size classes for the four deletion sequences and uniform distribution. The stars directly above the error bars denote significant differences in evenness between the focal deletion orders and the control group (uniform), detected using LME and Tukey post hoc test at 0.05 level of significance. Significant differences in the evenness of the four deletion orders are indicated by stars on lines connecting the compared indices: ********p* < 0.001; *******p* < 0.01; * *p* < 0.05; and NS, not significant.

**Supplementary References:**

Banašek-Richter C., Bersier L., Cattin M., Baltensperger R., Gabriel J., Merz Y., Ulanowicz R.E., Tavares A.F., Williams D.D. & Ruiter P.C. (2009). Complexity in quantitative food webs. *Ecology*, 90, 1470-1477.

Butler J.L., Gotelli N.J. & Ellison A.M. (2008). Linking the brown and green: nutrient transformation and fate in the Sarracenia microecosystem. *Ecology*, 89, 898-904.

Rooney N., McCann K.S. & Moore J.C. (2008). A landscape theory for food web architecture. *Ecol. Lett.*, 11, 867-881.
